# Neuronal glutathione loss leads to neurodegeneration involving gasdermin activation

**DOI:** 10.1038/s41598-023-27653-w

**Published:** 2023-01-20

**Authors:** Shoko Hashimoto, Yukio Matsuba, Mika Takahashi, Naoko Kamano, Naoto Watamura, Hiroki Sasaguri, Yuhei Takado, Yoshihiro Yoshihara, Takashi Saito, Takaomi C. Saido

**Affiliations:** 1 Laboratory for Proteolytic Neuroscience, RIKEN Center for Brain Science, 2-1 Hirosawa, Wako, Saitama 351-0198 Japan; 2 Pioneering Research Division, Medical Innovation Research Center, Shiga University of Medical Science, Seta Tsukinowa-Cho, Otsu, Shiga 520-2192 Japan; 3 Dementia Pathophysiology Collaboration Unit, RIKEN Center for Brain Science, 2-1 Hirosawa, Wako, Saitama 351-0198 Japan; 4 Department of Functional Brain Imaging, National Institutes for Quantum Science and Technology, 4-9-1 Anagawa, Inage-Ku, Chiba, 263-8555 Japan; 5 Laboratory for Systems Molecular Ethology, RIKEN Center for Brain Science, 2-1 Hirosawa, Wako, Saitama 351-0198 Japan; 6 Department of Neurocognitive Science, Institute of Brain Science, Nagoya City University Graduate School of Medical Sciences, 1 Kawasumi, Mizuho-Cho, Mizuho-Ku, Nagoya, 467-8601 Japan; 7 Department of Neuroscience and Pathobiology, Research Institute of Environmental Medicine, Nagoya University, Furo-Cho, Chikusa-Ku, Nagoya, Aichi 464-8601 Japan

**Keywords:** Cell death in the nervous system, Diseases of the nervous system, Neurological disorders

## Abstract

Accumulating evidence suggests that glutathione loss is closely associated with the progression of neurodegenerative disorders. Here, we found that the neuronal conditional-knockout (KO) of glutamyl-cysteine-ligase catalytic-subunit (GCLC), a rate-limiting enzyme for glutathione synthesis, induced brain atrophy accompanied by neuronal loss and neuroinflammation. GCLC-KO mice showed activation of C1q, which triggers engulfment of neurons by microglia, and disease-associated-microglia (DAM), suggesting that activation of microglia is linked to the neuronal loss. Furthermore, gasdermins, which regulate inflammatory form of cell death, were upregulated in the brains of GCLC-KO mice, suggesting the contribution of pyroptosis to neuronal cell death in these animals. In particular, GSDME-deficiency significantly attenuated the hippocampal atrophy and changed levels of DAM markers in GCLC-KO mice. Finally, we found that the expression of GCLC was decreased around amyloid plaques in *App*^*NL-G-F*^ AD model mice. *App*^*NL-G-F*^ mouse also exhibited inflammatory events similar to GCLC-KO mouse. We propose a mechanism by which a vicious cycle of oxidative stress and neuroinflammation enhances neurodegenerative processes. Furthermore, GCLC-KO mouse will serve as a useful tool to investigate the molecular mechanisms underlying neurodegeneration and in the development of new treatment strategies to address neurodegenerative diseases.

## Introduction

Oxidative stress has been suggested as a possible etiology in a number of neurodegenerative diseases, including Alzheimer’s disease (AD), Parkinson's disease (PD) and amyotrophic lateral sclerosis (ALS)^1^. Several events seen in the brain of neurodegenerative diseases could give rise to an abnormal production of reactive oxygen spices (ROS). For example, many studies observed mitochondrial damage in postmortem brain samples and mouse models of AD, PD and ALS^2,3^. While mitochondria produce ROS during oxidative phosphorylation, the ROS are appropriately eliminated by several antioxidants under normal physiological conditions. However, mitochondrial dysfunction can lead to a buildup of ROS due to an imbalance in their production and degradation^4,5^ Another major source of ROS is NADPH oxidase (Nox), a membrane-bound enzyme complex that produces superoxide by transferring an electron to oxygen from NADPH. One of the important roles of Nox-derived superoxide is killing foreign bacteria in the animal body in immune response. Nox activity also contributes to glial inflammatory response in the brain^6^. Under homeostatic physiological conditions, activated microglia and astrocytes can produce ROS and nitric oxide (NO) molecules that play roles in defense mechanisms against microbial pathogens. However, in the brain of neurodegenerative diseases, the excess production of ROS and NO results in neuronal damage^7–9^.

Further to the above, physiological processes that degrade ROS are known to be impaired in neurodegenerative diseases and as a consequence of aging. Glutathione is a master antioxidant produced in the cytoplasm and is the most abundant thiol in animal cells. Because the brain consumes a large amount of oxygen and leads to high production of reactive oxygen species, the antioxidative capacity of glutathione is important to keep brain homeostasis. However, the glutathione levels are altered in the brains of neurodegenerative diseases and with age. In AD, Mandal et al.^10,11^ demonstrated that GSH levels were significantly decreased in the frontal cortex and hippocampus of AD and mild cognitive impairment (MCI) patient groups compared with levels in an age-matched control group. In ALS, lower levels of GSH were found in the motor cortex and the corticospinal tractus in patients compared to healthy controls^12–15^. Lower levels of GSH were also observed in the substantia nigra of PD patients and the caudate nucleus of progressive supranuclear palsy (PSP) patients^16^. Moreover, Emir et al.^17^ showed lower glutathione levels in midsagittal sections of the optic lobe of elderly compared to young subjects. These findings suggest that glutathione dysfunction may be closely associated with neurodegenerative processes.

In the present study, we investigated the effects of glutathione loss-induced oxidative stress on brain pathologies. Glutathione biosynthesis is facilitated by a rate-limiting enzyme, glutamate-cysteine ligase (GCL), which consists of a catalytic subunit (GCLC) and a modifier subunit (GCLM). To elucidate the effect of GCLC and glutathione loss on brain homeostasis, we biochemically and histochemically analyzed brain pathologies in neuronal GCLC-conditional knockout (GCLC^floxed^ X CaMKII-Cre; GCLC-KO) mice and observed progressive neurodegeneration and marked neuroinflammation in the brains of these animals. Moreover, we demonstrated that neuroinflammatory mechanisms including gasdermin-mediated pyroptosis play important roles in the neurodegenerative process in GCLC-KO mice.

## Results

### Neuronal GCLC knockout leads to brain atrophy accompanied by neuronal cell death

To examine how the declines in GCLC and glutathione might affect brain homeostasis, we prepared GCLC^floxed^ X CaMKII-Cre (GCLC-KO) mice whose GCLC expression is deleted in calcium/calmodulin-dependent protein kinase II (CaMKII)-positive neurons. We confirmed that GCLC-KO mice have significantly reduced GCLC and glutathione levels, and an elevated ratio of oxidized to total glutathione in their brains (Fig. S1, Fig. S2A and Fig. S3).

**Figure 1:**
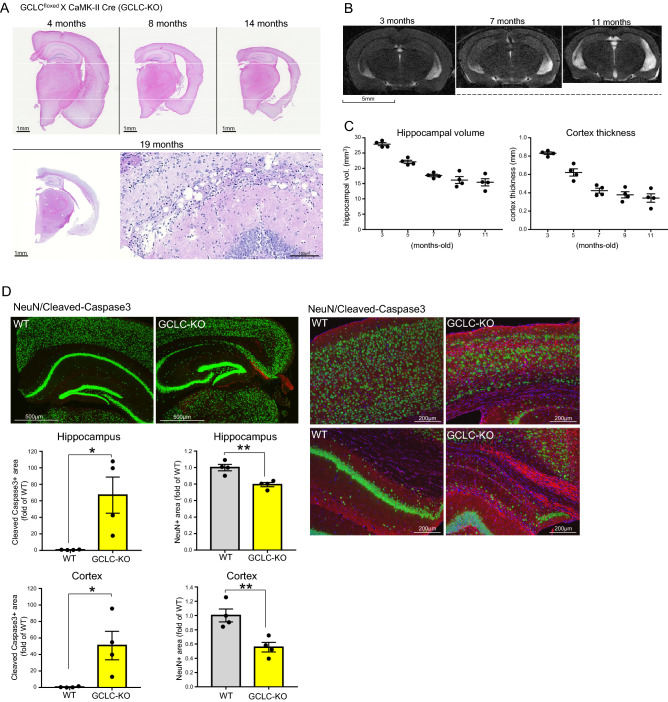
Neuronal GCLC-deficiency leads to brain atrophy due to neuronal loss. (**A**) Brain sections from 4-, 8-, 14- and 19-month-old GCLC-KO were stained by H&E. Lower right panel shows a magnified image of a 19-month-old mouse brain. (**B**) Representative MRI scans of GCLC-KO mouse brains scanned every 2 months from 3 to 11 months of age. (**C**) Values shown in the graph represent the mean bilateral hippocampal volume calculated from 8–9 scanned MR images (left) and cortical thickness measured from several images (right) ± SEM (n = 4; *p < 0.05, **p < 0.01). (**D**) Brain sections of 8-month-old GCLC-KO and WT were immunostained for NeuN (green)/cleaved-caspase 3 (red). Right panels were confocal MIP images of cortex (Upper panels) and hippocampal CA1(Lower panels) region. Values shown in the graphs are the fluorescence intensity of NeuN and caspase 3 with the results expressed as the mean relative levels ± SEM (n = 4; *p < 0.05, **p < 0.01).

Notably, we found age-dependent brain atrophy in GCLC^floxed^ X CaMKII-Cre mice (GCLC-KO) mice but not in wild-type (WT) mice (Fig. 1A, Fig. S2C). Vacuole-like degeneration was also observed in 19-month-old mice (Fig. 1A). The brain atrophy of GCLC-KO mice was also evidenced by MRI analyses (Fig. 1B,C, Fig. S2B and Fig. S4). Age-dependent declines were detected both in hippocampal volume and cortical thickness (Fig. 1B,C). Since the brain atrophy progressed as mice aged (Fig. 1A–C), the reduced brain volume was not due to developmental abnormality but to acquired neurodegeneration. We also detected a loss of the neuronal cell marker NeuN and an activation of cleaved caspase-3 (Fig. 1D), indicating that the brain atrophy in GCLC-KO was associated with neuronal loss. The neuronal loss and atrophy were particularly evident in the CA1 region of the hippocampus and layers II-III of the cerebral cortex. Similar pathological findings were observed in GCLC^floxed^ X Synapsin-Cre mice by Feng et al.^18^, who also reported behavioral disorders accompanying brain atrophy. Fernandez-Fernandez et al.^19^ has also reported dendrite disruption in hippocampal CA1 region and behavioral disorders in neuron-specific GCL knockdown mouse. In addition, there was no significant difference in the degree of neuronal loss between male and female GCLC-KO mouse (Fig. S5).

No brain atrophy was evident until animals reached at least until 3 months of age for GCLC^floxed^ X Iba1-Cre (Fig. S6 and Fig. S7A,D) mice and 8 months of age for GCLC^floxed^ X GFAP-Cre mice (Fig. S7A–C). Because GCLC^floxed^ X Iba1-Cre mice died early (around 3–4 months of age) due to developmental defects, we could not assess the effect of microglial GCLC-deficiency on brain atrophy. These results suggest that the decline in glutathione in neurons is more critical for the brain atrophy than that in astrocytes.

### The GCLC-KO mouse brain displays severe neuroinflammation at an early stage of brain atrophy

**Figure 2:**
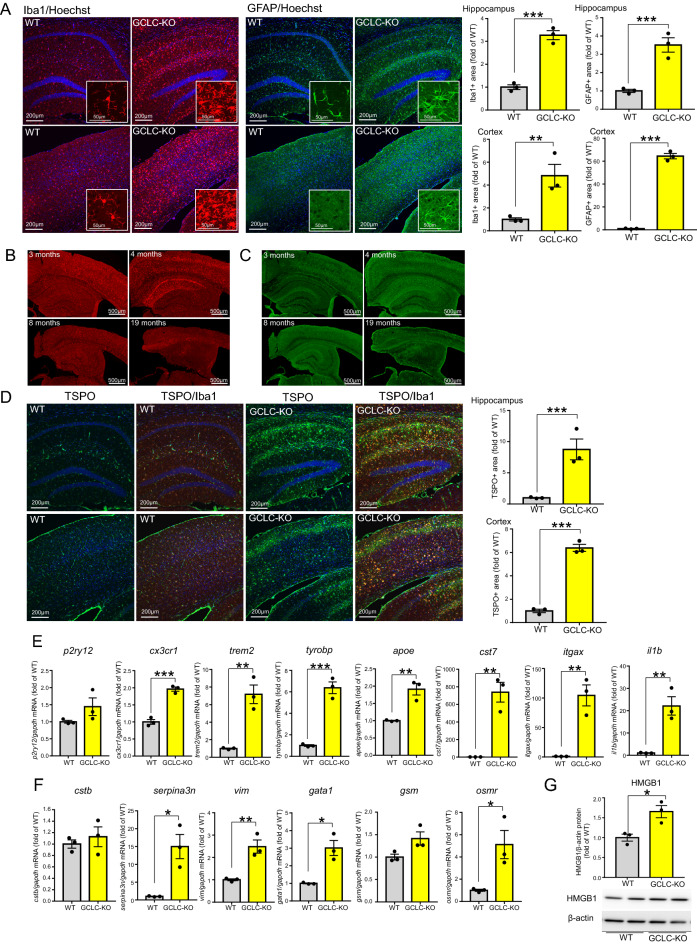
GCLC deficiency induces severe neuroinflammation. (**A**) Brain sections of 5-month-old WT (each left panel) and GCLC-KO (each right panel) were immunostained using Iba1 (left, red) or GFAP (right, Green) antibody and stained with Hoechst (Blue). Upper panels show hippocampal CA1 region, and lower panels show cortical region. Values shown in the graphs represent the mean intensity level ± SEM (n = 3; **p < 0.01, ***p < 0.001). (**B,C**) Brain sections of 3-,4-,8- and 19-month-old GCLC-KO mice were immunostained with Iba1 (**B**) or GFAP (**C**) antibody. (**D**) Representative images of brain sections from 5-month-old WT and GCLC-KO immunostained with TSPO (Green) and Iba1 (red) antibodies. Upper panels show hippocampal CA1 region, and lower panels show cortical region. Values shown in the graphs represent the mean intensity level ± SEM (n = 3; ***p < 0.001). (**E,F**) mRNA levels of microglial (**E**) and astrocyte (**F**) markers in 4-month-old WT and GCLC-KO were determined by qRT-PCR. Values shown in the graphs represent the mean relative expression level ± SEM (n = 3; *p < 0.05, **p < 0.01, ***p < 0.001). (**G**) Tris–HCl soluble fractions were prepared from the cortices of 4-month-old WT and GCLC-KO. HMGB1 protein levels were determined. Values shown in the graph represent relative expression level of HMGB1 (n = 3; *p < 0.05).

Numerous studies have noted the importance of neuroinflammation in neurodegenerative processes^20^. We therefore examined if neurodegeneration in the GCLC-KO mouse is associated with neuroinflammation. Immunostaining of brain tissue for Iba1 and GFAP revealed that microglia and astrocytes were markedly activated in GCLC-KO mouse brains (Fig. 2A). Similar findings were also observed in neuron-specific GCL knockdown mouse by Fernandez-Fernandez et al.^19^. The activation of glial cells was observed in similar regions to those where neuronal loss and atrophy were severe, i.e. the CA1 region of the hippocampus and layers II-III of the cerebral cortex. Numbers of microglia and astrocytes peaked in mice at 4 months of age when brain atrophy had just commenced (Fig. 2B,C, Fig. S8A–D). We also observed an increase in the 18 kDa translocator protein TSPO, which is a mitochondrial cholesterol-binding protein and upregulated in microglia during inflammatory activation associated with neurodegeneration (Fig. 2D). These results indicate that neuroinflammation is strongly associated with neuronal death in GCLC-KO mice, particularly in the early stages of brain atrophy.

Recently, single-cell (sc) RNA sequence (RNA-seq) analyses of immune cells from brain samples of an AD model (5XFAD) identified microglial clusters named disease-associated microglia (DAM), which appear under neurodegenerative conditions^21^. Microglia are initially activated to stage 1 DAM, which are characterized by a downregulation of homeostatic microglial genes including *p2ry12* and *cx3cr1*, and an upregulation of genes including *trem2*, *tyrobp* and *apoe*. The activated Trem2 signal in stage 1 DAM facilitates transition to stage 2 DAM, which is characterized by an upregulation of lysosomal, phagocytic, and lipid metabolism pathway-related genes including *cst7* and *itgax*. Similarly, scRNA-seq analyses of 5XFAD mice also identified disease-associated astrocyte (DAA) clusters related to AD and aging conditions, in which genes including *cstb*, *serpina3n*, *vim*, *gata1*, *gsm*, and *osmr* were highly expressed^22^. To investigate whether DAM and DAA are associated with neurodegeneration in GCLC-KO mice, we conducted qRT-PCR analyses of DAM and DAA-related genes using cortical samples from 4-month-old GCLC-KO and WT mice (Fig. 2E,F). We observed higher levels of stage 1 and 2 DAM marker genes, the inflammatory marker *il1b* (Fig. 2E), and DAA marker genes except for *cstb* and *gsm* (Fig. 2F). These results indicate that the activation of microglia and astrocytes into DAM and DAA is involved in neurodegeneration in GCLC-KO mice. On the other hand, we could not detect any downregulation of microglial homeostatic genes (*p2ry12* and *cx3cr1*), possibly because it was difficult to detect differences in qRT-PCR using bulk samples. Microglia are transformed into DAM by recognizing Neurodegeneration-Associated Molecular Pattern (NAMP) danger signals which leak from damaged neuronal cells in analogy to the peripheral immune system’s pathogen- and damage-associated stress signals (PAMPs and DAMPs)^23^. As a representative molecule of NAMPs/DAMPs/PAMPs, the nuclear protein high-mobility group box protein 1 (HMGB1) plays an important role in CNS-related pathologies by being released from damaged cells^24^. HMGB1 in the soluble fraction of detergent-free buffer was elevated in GCLC-KO mouse brain tissue (Fig. 2G), suggesting that danger signal molecules from damaged neurons lead to microglial and astrocytic activation.

### Microglial and astrocytic proteins are preferentially elevated in the GCLC-KO mouse brain

**Figure 3:**
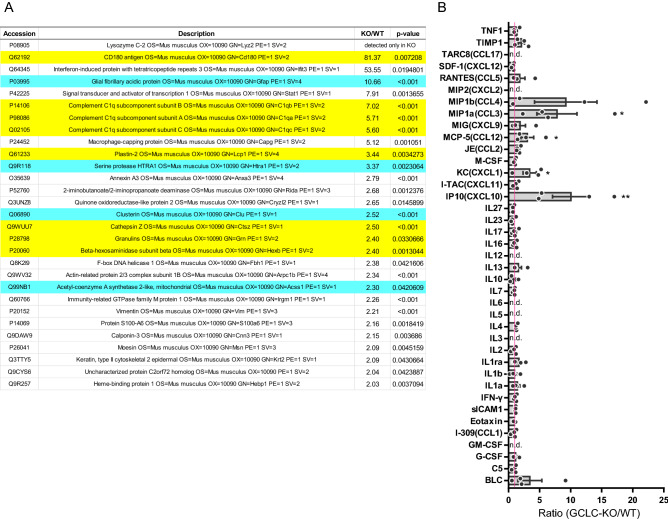
Comprehensive analyses of GCLC-KO mouse brains. (**A**) Proteomic analyses were performed using protein samples from the cortices of 4-month-old WT and GCLC-KO. Values shown in the table represent the relative expression level of each protein in GCLC-KO/WT. Proteins whose expression levels were > twofold greater in GCLC-KO, in addition to being significant at the *p* < 0.05 level, are listed. Yellow and cyan highlights proteins whose mRNA are abundant in microglia or astrocytes, respectively. The mRNA expression of each protein was referred to Mousebrain.org (http://mousebrain.org/genesearch.html). (**B**) Protein levels of cytokines in 4-month-old WT and GCLC-KO were determined by cytokine array. Values shown in the graph represent the mean relative expression level of cytokines in GCLC-KO/WT (n = 4; *p < 0.05).

To uncover mechanisms leading to brain atrophy in GCLC-KO mice, we next performed a comprehensive analysis of protein expression by Liquid Chromatography-Mass spectrometry (LC–MS) analysis using brain samples from 4-month-old GCLC-KO mice (Fig. 3A and Extended Data file). Here, approximate levels of proteins were determined using the abundance of identified peptides without labeling such as^18^ O or iTRAQ. Figure 3A shows proteins whose expression levels were > twofold greater in GCLC-KO vs WT, with differences significant at the *p* < 0.05 level. Yellow and cyan highlights show proteins for which mRNA are abundant in the microglia and astrocytes, respectively. As expected, the list included numerous microglial- and astrocyte-related. We detected upregulation of INF-STAT1 pathway-related proteins (IFIT3 and STAT1), taking into account that upregulation of the *ifit3* gene has been seen in a cluster of microglia named interferon response microglia (IRM) as determined by scRNA-seq analyses in the *App*^*NL-G-F*^ mouse^25^. Remarkably, we found dramatic increases of C1q isoforms, which are involved in synapse pruning by microglia, suggesting that C1q-mediated phagocytosis by the microglia is facilitated in GCLC-KO mice.

Under inflammatory conditions, glial cells release cytokines and chemokines, many of which are associated with cell death signaling. We therefore examined released cytokines and chemokines in GCLC-KO mice using a cytokine array kit from R&D Systems. Figure 3B shows levels of cytokines in GCLC-KO mice relative to WT mice, and indicates the upregulation of several cytokines and chemokines in GCLC-KO mice. Among these chemokines, elevations in CCL3, CCL4 and CXCL10 were marked (Fig. 3B). CCL3 and CCL4 are produced by microglia and astrocytes, and their upregulation is observed in postmortem brains from AD patients^26,27^. In addition, CCL3 production by astrocytes, which is associated with the ApoE genotype^28^, impairs synaptic transmission and plasticity in the mouse hippocampus^29^, suggesting a strong relationship between CCL3 activation and AD pathologies. CXCL10 is mainly released from astrocytes^27^, and is markedly elevated in the AD brain^30^. CXCL10 and its receptor (CXCR3) regulate microglial activation and recruitment^31,32^, and were shown to promote plaque formation in an AD mouse model^33^. Correspondingly, these cytokines should mediate microglial activation and neuronal damage in GCLC-KO mice.

### C1q-mediated microglial phagocytosis is involved in the neuronal loss seen in GCLC-KO mice

**Figure 4:**
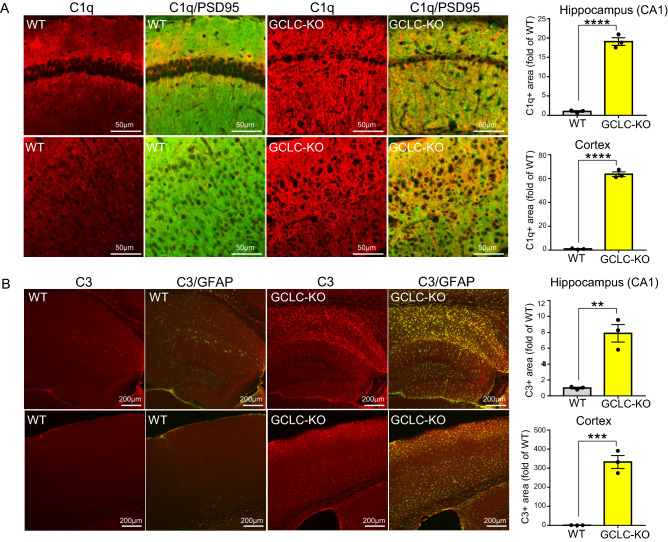
Complements are activated in GCLC-KO mouse brains. (**A,B**) Brain sections from 5-month-old WT (left panels) and GCLC-KO (right panels) were immunostained using C1q (Red) and PSD95 (neuronal cell marker) (Green) antibodies (**A**) or C3 (Red) and GFAP (Green) antibodies (**B**). Upper panels show the hippocampal CA1 region, and lower panels show the cortical region. Values shown in the graphs represent the mean intensity level ± SEM (n = 3; **p < 0.01, ***p < 0.001, ****p < 0.0001).

A proteomic analysis of GCLC-KO revealed the upregulation of C1q (Fig. 3A). Complements C1q and C3 participate in synapses and apoptotic cells elimination through microglial-mediated phagocytosis by serving as “eat-me signals” on synapses and apoptotic cells that trigger microglia to recognize and engulf them^34,35^. This system is associated with synapse and neuronal loss in various neurodegenerative disorders^34,36^. To validate the activation of complements in GCLC-KO, we carried out immunohistochemical analyses to detect C1q and C3 in brain tissue of GCLC-KO mice. Consistent with the proteomic data described above, we detected significantly higher levels of C1q in GCLC-KO compared with WT mice (Fig. 4A). Notably, the upregulated C1q signal overlapped with a neuronal cell marker signal (PSD95), suggesting that C1q exists in neurons and acts as an “eat me signal”. C3 was also upregulated in GCLC-KO mouse brain tissue, and primarily in astrocytes where C3 is mainly produced (Fig. 4B).

**Figure 5:**
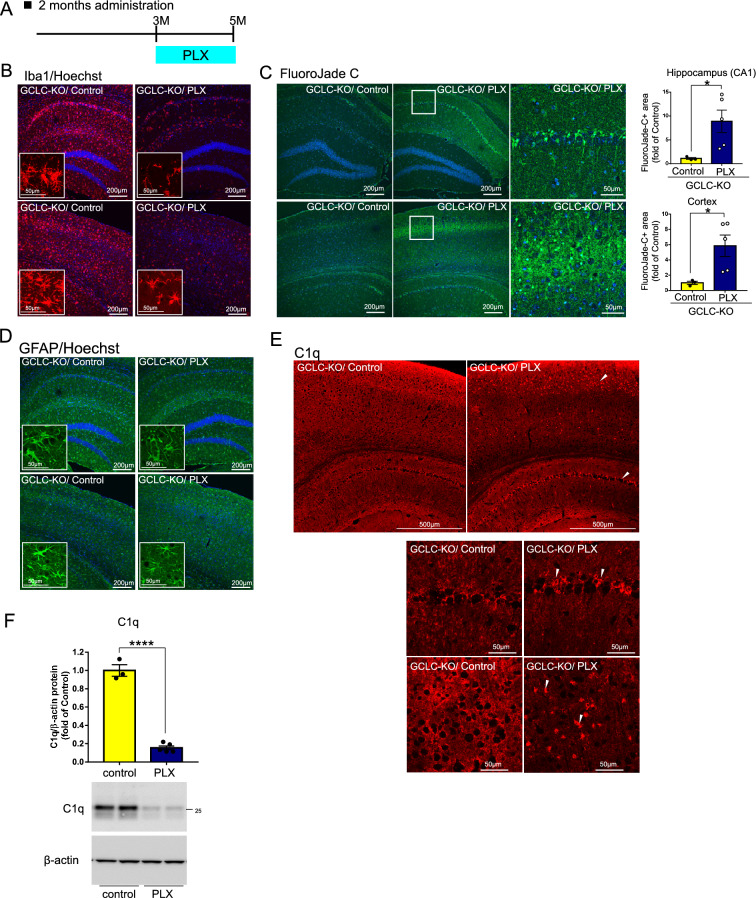
Synaptic pruning by C1q is involved in the reduced volume of GCLC-KO mouse brains. (**A**) Scheme showing PLX3397 (PLX) administration to mice for 2 months. GCLC-KO mice were fed a PLX3397 (PLX)-mixed diet or control diet for 2 months (from 3 to 5 months of age), and brain samples were collected at 5 months of age. (**B–E**) Brain sections from PLX- or control-chow treated GCLC-KO mice were immunostained with Iba1 antibody (**B**), stained with FluoroJade C (**C**), immunostained with GFAP antibody (**D**) or immunostained with C1q antibody (**E**). (**B–D**) Upper panels show the hippocampal CA1 region, and lower panels show the cortical region. (**C**) Magnified images of right panels show the hippocampal CA1 region (upper panels) and layers II–III of the cerebral cortex (lower panels) of PLX treated GCLC-KO mouse. Values shown in the graphs represent the mean intensity level ± SEM (n = 3; **p < 0.01). (**E**) Magnified images of lower panels show the hippocampal CA1 region (upper panels) and layers II-III of the cerebral cortex (lower panels). (**F**) C1q protein levels in the brains of PLX-treated or control mice were determined by western blotting. Values shown in the graph represent relative expression level of C1q (n = 3 (control) or 5 (PLX); ****p < 0.0001).

To investigate the contribution of microglial phagocytosis to neuronal loss, we next tested whether microglial elimination using PLX3397 (PLX), a colony-stimulating factor 1 receptor (CSF1R) inhibitor, affected brain pathology in GCLC-KO mice. In a first experiment, 3-month-old GCLC-KO or WT mice were fed for 2 months with PLX formulated-chow and brain samples were collected at the end of this period (Fig. 5A). We confirmed that the PLX treatment significantly decreased the number of microglia, though the removal was not complete (Fig. 5B). Astrocyte was slightly increased by PLX treatment (Fig. 5D). Intriguingly, PLX-treated GCLC-KO showed a higher level of FluoroJade C signal, which stains degenerating neurons^37^ (Fig. 5C). Since the FluoroJade C signal was not detected in PLX-treated WT mice (Fig. S8E,F), this suggests that FluoroJade C did not react with microglia killed by PLX but rather with damaged neurons. These results indicate that damaged neurons escaped being engulfed due to microglia depletion, and the remaining damaged cells were stained with FluoroJade C in PLX-treated GCLC-KO mice. Further to these findings, we observed intense C1q signals in hippocampal pyramidal cell layer and cortical layers II-III from PLX-treated GCLC-KO mice (arrowheads in Fig. 5E). While the total level of C1q was decreased by the depletion of microglia – the main source of C1q (Fig. 5F) – it strongly accumulated in some cells (Fig. 5E) in such a way that accumulated C1q may serve as a tag for damaged neuronal cells so that microglia find and phagocytose these cells. Taken together, phagocytosis of damaged neurons by microglia may lead to brain atrophy in GCLC-KO mice, with complement proteins playing important roles in the phagocytic process.

### Gasdermin-mediated pyroptosis participates in the neurodegeneration of GCLC-KO mice

We previously found that cleavage of gasdermin (GSDM) D and GSDME were facilitated in CAPON (c-terminal PDZ ligand of nNOS)-overexpressing mice^38^. In these animals, the CAPON gene is introduced into the brain by an adeno-associated virus and the brain displays progressive neurodegeneration^38^. Gasdermin family proteins display membrane pore-forming activity and facilitate the secretion of humoral factors. GSDMD acts as an essential effector of pyroptosis by transmitting cytokines and chemokines from secreting cells. GSDME is known to be activated by caspase-3-mediated cleavage under apoptotic conditions, with the activation accelerating pyroptosis and necroptosis. Our previous findings suggest that the activity of GSDMD and GSDME could be profoundly involved in the process of neurodegeneration.

**Figure 6:**
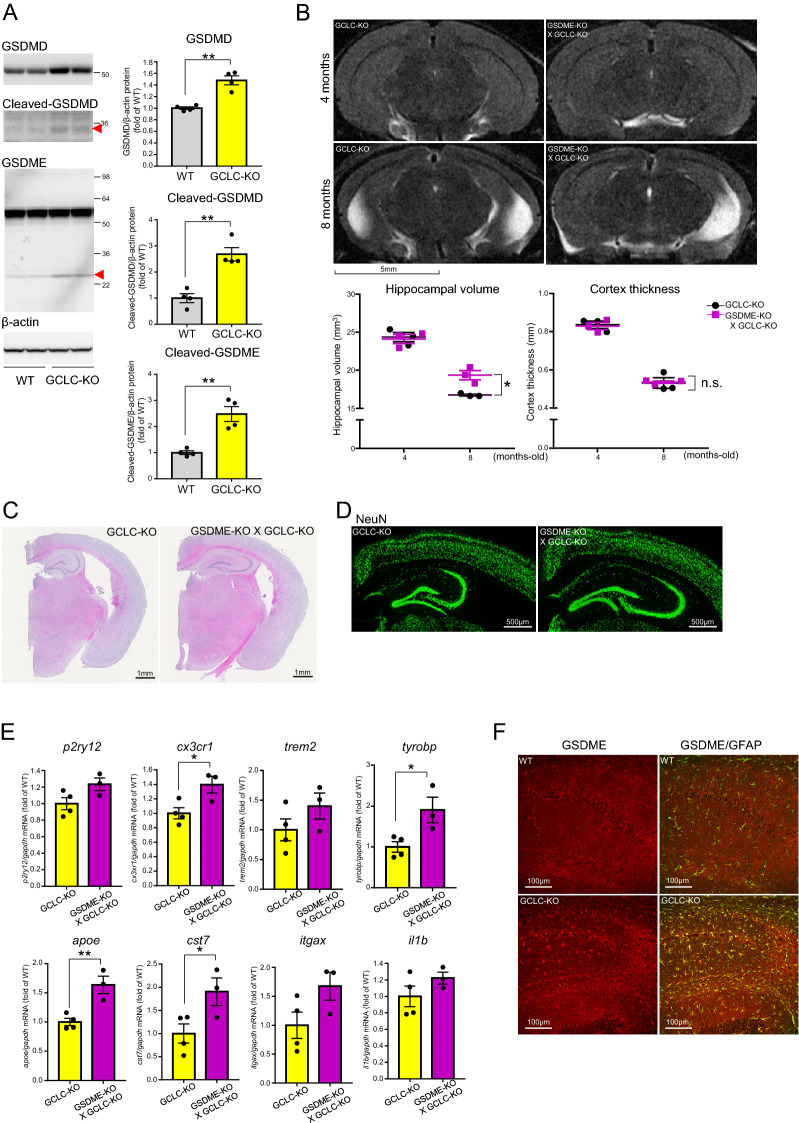
GSDME plays an important role in the neurodegeneration. (**A**) GSDMD and GSDME protein levels in 8-month-old WT and GCLC-KO mouse brains were determined by western blotting. Red arrowheads show cleaved form (active form) of GSDMD and GSDME, respectively. Values shown in the graph represent relative expression level of each protein (n = 3; **p < 0.01). (**B**) Representative MRI scans of GSDME-KO X GCLC-KO and GCLC-KO mouse brains scanned at 4 and 8 months of age. Values shown in the graph represent the mean bilateral hippocampal volume (left) and cortical thickness (right) ± SEM. Differences between groups were analyzed by two-way ANOVA (n = 3; *p < 0.05). (**C**) Brain sections of 8-month-old GSDME-KO X GCLC-KO, GSDMD-KO X GCLC-KO and GCLC-KO were stained by H&E. (**D**) Brain sections of 8-month-old GSDME-KO X GCLC-KO and GCLC-KO were immunostained with NeuN antibody. (**E**) mRNA levels of microglia markers in GSDME-KO X GCLC-KO and GCLC-KO were determined. Values shown in the graph represent the mean relative expression level ± SEM (n = 3; *p < 0.05, **p < 0.01). (**F**) Brain sections of 5-month-old WT and GCLC-KO were immunostained with GSDME and GFAP antibodies.

Since GCLC-KO mice also show progressive neurodegeneration and secretion of inflammatory chemokines, we predicted that GSDMD and GSDME might contribute to the neuronal death seen in these mice. Indeed, we observed an increase in both the precursor and mature forms of GSDMD and in the mature form of GSDME (Fig. 6A), indicating that gasdermin protein-mediated pyroptosis and necroptosis could be involved in neuronal death in GCLC-KO mice. To further investigate the roles of GSDMD and GSDME in neurodegeneration, we analyzed brain pathologies in GSDMD- or GSDME-KO X GCLC-KO (Fig. S10A) mice. Interestingly, GSDME deficiency significantly attenuated the hippocampal atrophy seen in GCLC-KO mice (Fig. 6B–D), while GSDME-single-KO mice showed no change in hippocampal volume compared to WT (Fig. S11A). On the other hand, the GSDMD deficiency did not significantly change the degree of atrophy (Fig. S9A). Further to this, we examined expression levels of microglial and astrocytic markers in GSDME-KO X GCLC-KO mice and found that the expression of several DAM-related genes was significantly enhanced in 4-month-old mice compared to age-matched single GCLC-KO mice (Fig. 6E). DAA markers were not affected by the GSDME deficiency (Fig. S10B). Enhancement of DAM-related genes was not determined in GSDMD X GCLC-KO mice (Fig. S9B) and GSDME-single-KO mice (Fig. S11B). From these results, it could be postulated that GSDME contributes to the conversion of microglia by mediating the secretion of molecules that downregulate DAM activation, and that protective roles of DAM could be involved in the reversal of neurodegeneration.

GSDME has been extensively studied in the field of cancer biology; however, its function in the central nervous system has yet to be elucidated. To understand where GSDME is activated in GCLC-KO mouse brain, we performed GSDME immunohistochemistry in 8-month-old GCLC-KO mice (Fig. 6F). While GSDMD signals were observed in microglia (Fig. S9C), GSDME signals were in astrocytes (Fig. 6F) which is consistent with GSDME participating in the secretion of astrocytic factors. Many reports have demonstrated communication between astrocytes and microglia via the secretion of factors that regulate their physiological state^39,40^. We actually performed cytokine array using protein sample of GSDME-KO X GCLC-KO mouse. Within 5 cytokines/chemokine that tended to be increased in GCLC-KO, KC (CXCL1) and MIP1b (CCL4) were lowered in GSDME-KO X GCLC-KO compared with GCLC-KO (Fig. S10C). These chemokines could be involved in GSDME-mediated interactions between astrocyte and microglia. We consider that GSDME is involved in the secretion of astrocytic factors which may regulate microglial activation.

### *App*^*NL-G-F*^ mouse exhibits inflammatory events similar to that of GCLC-KO mouse

**Figure 7:**
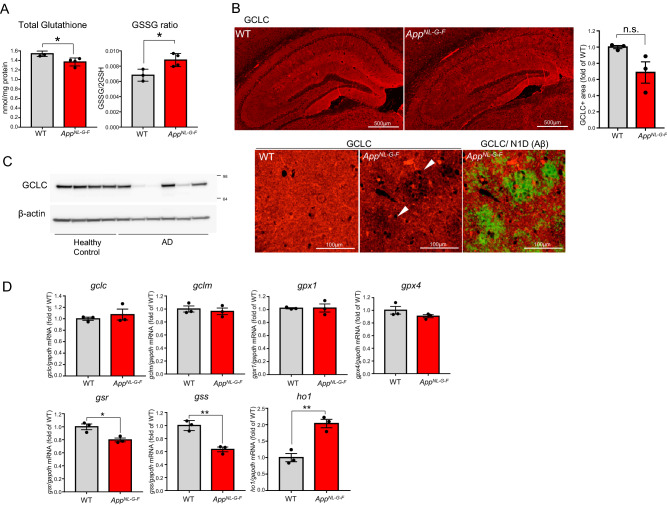
Glutathione homeostasis is disrupted in the presence of amyloid pathology. (**A**) Total glutathione levels (left) and GSSG (oxidized form) to GSH ratio (right) in the cortices of 18-month-old WT and *App*^*NL-G-F*^ were determined. Values shown in the graph represent nmol/mg protein (total levels) or ratio of GSSG/2GSH (GSSG ratio) expressed as the mean level ± SEM (n = 3; *p < 0.05). (**B**) Brain sections of 24-month-old wild type (WT) and *App*^*NL-G-F*^ were immunostained with GCLC antibody (upper panels). Fluorescence intensity in the hippocampus is quantitatively represented as the mean intensity level ± SEM (n = 3). A magnified image of *App*^*NL-G-F*^ showing double-staining for GCLC antibody and Aβ antibody (N1D) (lower panels). (**C**) GCLC protein levels were detected in cortical samples from AD patients and healthy controls. Details of the human samples are provided in Watamura et al.^66^ (**D**) mRNA levels of glutathione-related genes in 12-month-old WT and *App*^*NL-G-F*^ were determined. Values shown in the graphs represent the mean relative expression level ± SEM (n = 3; *p < 0.05, **p < 0.01).

We then asked whether the mouse model of neurodegenerative diseases showed inflammatory events that we had seen in GCLC-KO mice. Here, we investigated glutathione homeostasis and inflammatory events in *App*^*NL-G-F*^ knockin AD mouse model. We first examined whether glutathione levels were also altered in *App*^*NL-G-F*^ knockin (*App*^*NL-G-F*^ ) mice where amyloid pathology appears by the age of 2–3 months^41^. Glutathione levels in the brains of these mice were significantly lower and the redox state was more oxidized compared with wild type (WT) controls (Fig. 7A). This result is consistent with reports by Izumi et al. and Uruno et al.^42,43^ who observed lower GSH levels in the brains of *App*^*NL-G-F*^ mice. A rate-limiting step in the glutathione synthesis pathway is mediated by GCL which comprises a catalytic subunit (GCLC) and a modifier subunit (GCLM). We subsequently determined GCLC levels in *App*^*NL-G-F*^ mouse brains and WT controls by immunohistochemistry (Fig. 7B and Fig. S12). While there were no significant differences in total GCLC levels between WT and *App*^*NL-G-F*^ (Fig. 7B), depletion of GCLC was observed around amyloid beta (Aβ) plaques in *App*^*NL-G-F*^ mice (Fig. 7B). GCLC signals were reduced associated with reduction of post synaptic markers around amyloid plaques (Fig. S12). In addition, a decline in GCLC was detected in some human brain samples with AD (Fig. 7C).

We next examined mRNA levels of GCLC and glutathione homeostasis-related genes (Fig. 7D). *App*^*NL-G-F*^ mice showed lower levels of glutathione reductase (*gsr*) and glutathione synthase (*gss*) (Fig. 7D), indicating that glutathione homeostasis was perturbed by the amyloid pathology. GSS decline is consistently observed in 3xTg-AD AD model mouse^44^ and in human brain tissues with AD^45^, suggesting relevance to AD pathogenesis. We also detected increased levels of an oxidative stress marker, hemeoxigenase1 (*ho1*), in the brains of *App*^*NL-G-F*^ mice.

**Figure 8:**
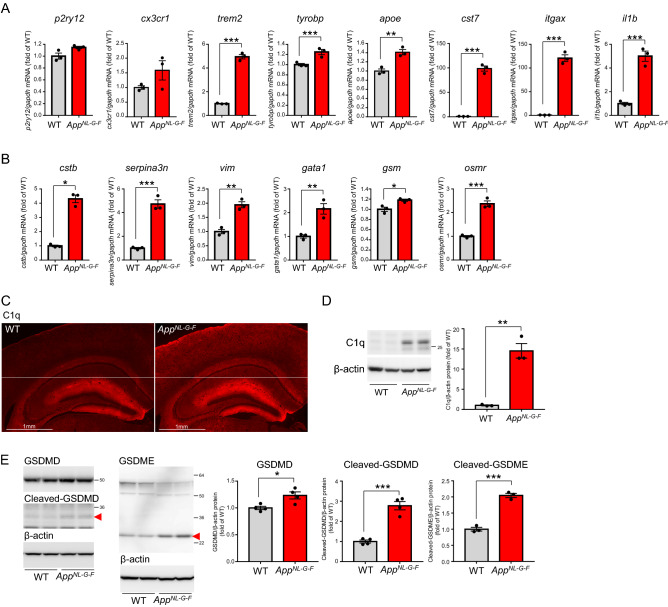
*App*^*NL-G-F*^ mouse shows inflammatory events similar to that of GCLC-KO mouse. (**A,B**) mRNA levels of microglial (**A**) and astrocyte (**B**) markers in 12-month-old WT and *App*^*NL-G-F*^ were determined. Values shown in the graphs represent the mean relative expression level ± SEM (n = 3; *p < 0.05, **p < 0.01, ***p < 0.001). (**C**) Brain sections from 6-month-old WT (left panels) and *App*^*NL-G-F*^ (right panels) were immunostained using C1q antibodies. (**D**) C1q protein levels in 6-month-old WT and *App*^*NL-G-F*^ were determined. Values shown in the graph represent relative expression levels of C1q (n = 3; **p < 0.01). (**E**) GSDMD and GSDME protein levels in 12-month-old WT and GCLC-KO mouse brains were determined. Red arrowheads show cleaved form (active form) of GSDMD and GSDME, respectively. Values shown in the graph represent relative expression level of each protein (n = 3 or 4; *p < 0.05, ***p < 0.001).

We next investigated whether *App*^*NL-G-F*^ mouse also exhibited inflammatory events that we had observed in GCLC-KO mice, i.e. induction of DAM and DAA-related genes, elevation of C1q, and activation of gasdermins. Sobue et al.^46^ have reported that *App*^*NL-G-F*^ mice express higher levels of DAM marker genes. We consistently observed that DAM-related genes were upregulated in *App*^*NL-G-F*^ mice compared with age-matched WT mice (Fig. 8A). Expression of DAA marker genes was also enhanced in *App*^*NL-G-F*^ mice (Fig. 8B). Moreover, we detected significant elevation of C1q (Fig. 8C,D) and activation of gasdermins (Fig. 8E) in *App*^*NL-G-F*^ mice. These results indicate that *App*^*NL-G-F*^ mice exhibit inflammatory events similar to that of GCLC-KO mice. Our observations are consistent with a report that mRNA expression of C1q and a part of DAM-related genes are upregulated associated with Aβ plaques in *App*^*NL-G-F*^ mouse^47^. Loss of glutathione due to amyloid pathology could be involved in such inflammatory responses in *App*^*NL-G-F*^ mouse. In addition, GCLC-KO mice have much in common neuroinflammatory mechanisms with neurodegenerative disease models including *App*^*NL-G-F*^, which means that GCLC-KO mouse will be a good tool to investigate the molecular mechanisms of neurodegeneration.

## Discussion

Many reports have revealed that glutathione loss and oxidative stress are closely associated with the progression of neurodegenerative disorders; however, little is known about the molecular mechanisms that link these processes. Here, we found that neuronal GCLC knockout leads to brain atrophy accompanied by neuronal cell death. Moreover, we have shown that neuroinflammation is strongly linked to neurodegeneration as a consequence of oxidative stress. To this end, we observed increases in cytokine and chemokine levels, an elevation of complement proteins that enhance microglial phagocytosis, an upregulation of DAM-related genes, and activation of pyroptosis via gasdermins in a GCLC-deficient mouse model. These findings suggest that a reduction in glutathione due to ageing and neuropathological progression are strongly involved in the ongoing progression of neurodegeneration. Accumulating evidence has demonstrated that oxidative stress due to neuroinflammation is accompanied by glial ROS production^7,48^, whereas our study has demonstrated that the reverse is also true. In other words, that neuroinflammation and oxidative stress activate each other, and the vicious cycle could lead to neurodegeneration.

Shih et al.^49^ demonstrated that activated astrocytes synthesized and released GSH to protect neuronal redox homeostasis. Vargas et al.^50^ showed that increased GSH synthesis in spinal cord astrocytes from ALS model rats inhibited motor neuron apoptosis. These findings suggest that astrocyte has a critical role in glutathione homeostasis in the central nervous system. On the other hand, we observed no brain atrophy in GCLC^floxed^ X GFAP-Cre mice, suggesting that astrocytes could make compensation for loss of glutathione in neurons upon neuronal damage.

We found that the cleavage of gasdermin proteins was facilitated in GCLC-KO mice. While GSDMD was localized to the microglia, GSDME accumulated in the astrocytes of GCLC-KO mice. Moreover, knockout of GSDME altered the microglial status and reduced atrophy in the hippocampus of GCLC-KO mice. From these results, we suggest that GSDMD and GSDME mediate cell–cell communication between microglia and astrocytes by accelerating the release of cytoplasmic contents. As the rescue of brain atrophy by GSDME knockout was only slight, we speculate that there is functional redundancy within the gasdermin family proteins. To date, few studies have focused on GSDME functions in the central nervous system; however, our study highlights the importance of GSDME-mediated signaling in the process of neuroinflammation and neurodegeneration. Indeed, we also detected gasdermin activation in CAPON-overexpressing mice, which show brain atrophy accompanying neuronal loss^38^. In addition, several studies have reported associations between genetic mutations in GSDME and hearing loss^51,52^. An abnormal activation of GSDME due to such mutations was considered to be the cause of cytotoxicity and the observed symptoms^53,54^. While GSDME functions in neurodegeneration and neuroinflammation are still to be fully elucidated, GSDME nevertheless shows promise as a key factor driving oxidative stress-induced neuronal cell death.

We observed an upregulated expression of DAM and DAA markers in GCLC-KO mouse brains, which aligns with a report by Uruno et al.^43^ who demonstrated that upregulation of stage 1 and 2 DAM markers in the *App*^*NL-G-F*^ mouse was attenuated by crossbreeding with *Keap1*^*FA/FA*^ mice, whose oxidative stress response is overactivated by genetically induced Nrf2. These findings suggest that oxidative stress is closely related to DAM induction. Sobue et al.^46^ suggested that a loss of homeostatic microglia is associated with neurodegeneration, because homeostatic genes including *p2ry12* were decreased in the microglia of tauopathy and motor neuron disease model mice accompanying neuronal loss, but not in the microglia of *App*^*NL-G-F*^ mice, which do not show neuronal loss. On the other hand, we observed no decline of *p2ry12* in our GCLC-KO mice, although neuronal loss was observed. It is possible that we did not detect such a decline because we used bulk samples in qRT-PCR; however, the decline of *p2ry12* may not be necessarily essential for neurodegeneration. Further analyses are needed to clarify mechanisms of glial activation under conditions of oxidative stress. A scRNA-seq analysis of the GCLC-KO mouse brain will provide further detail of glial function and diversification under oxidative stress and neurodegenerative conditions.

Synaptic pruning mediated by C1q has been identified in developmental, aging, and neurodegenerative processes in several diseases^34,55,56^. In the present study, C1q protein was increased in the GCLC-KO mouse brain and its neuronal accumulation was facilitated by microglial depletion, suggesting that C1q serves as a tag for damaged neurons by enabling them to be identified and engulfed by microglia. In addition to the function of C1q in phagocytosis, its elevated levels in microglia promote the secretion of pro-inflammatory cytokines, ROS, NO, and calcium^57,58^. This means that glutathione loss can give rise to a vicious cycle of C1q elevation and oxidative stress, with C1q being a key factor that leads to neurodegeneration under conditions of oxidative stress.

While glutathione was significantly depleted in GCLC-KO mouse brains (> 20% versus WT), the actual decrease of glutathione levels in AD brains is not so severe according to Mandal et al.^11^. However, a local drastic lowering or prolonged depression of glutathione under pathogenic conditions could gradually induce neuronal damage. In fact, local oxidative stress surrounding plaques has been observed in APP/PS1 mice by using redox-sensitive variants of green fluorescent protein (roGFP)^59^. While *App* knockin mouse shows no significant neurodegeneration and neuronal loss, it shows synaptic loss and reduction of a part of neuronal cells including active place cells around Aβ plaques^41,60^. Glutathione loss in neuron around Aβ plaques could be involved in such neuronal damages.

The decrease of glutathione levels has been detected in several AD mouse models including triple-transgenic (3X-AD-Tg) and APP/PS1 mouse^61,62^. On the other hand, glutathione-mimetic compounds or γ-glutamylcysteine (γ-GC), the limiting substrate for GSH biosynthesis, ameliorate brain pathologies and memory deficits in APP/PS1^63,64^. These findings also demonstrate the contribution of glutathione decrease to neuronal damage in AD.

In this study, we have experimentally demonstrated that glutathione loss-triggered oxidative stress can induce neurodegeneration, and that neuroinflammation plays an important role in the process of oxidative stress-induced neurodegeneration. In regard to treatments targeting oxidative stress, Izumi et al.^42^ demonstrated that glutathione administration attenuated inflammatory responses in *App*^*NL-G-F*^ knock-in mice, while Hongo et al.^65^ reported that astaxanthin, a carotenoid regarded as a highly potent antioxidant, ameliorated parvalbumin-positive neuron deficits and AD-related pathology in the hippocampi of *App*^*NL-G-F*^ mice. Therapeutic interventions in the vicious cycle of oxidative stress and neuroinflammation could offer a promising method for treating neurodegenerative diseases. Furthermore, the GCLC^floxed^ X CaMKII-Cre displays neurodegeneration without overexpressing pathology related genes. This mouse will be a useful tool to investigate the molecular mechanisms underlying neurodegeneration.

## Material and methods

### Animals

The GCLC^floxed^ mice were introduced from The European Mouse Mutant Archive (EMMA). The international strain name is “C57BL/6NTac-Gclctm1a(EUCOMM)Wtsi/WtsiCnbc”. The GCLC^floxed^ mice (Cre-negative) shows lowered levels of glutathione in blood probably because introduced *loxp* sequences interrupt GCLC expression (Fig. S3). The CaMKII-Cre mice were kindly provided by Shigeyoshi Itohara (RIKEN Center for Brain Science). The GCLC^floxed^ X CaMKII-Cre mouse was named here as “GCLC-KO” for the sake of convenience. GSDMD-KO (C57BL/6 N-Gsdmd^em4Fcw/^J; Stock No.032410) and GSDME-KO (C57BL/6 N-Gsdme^em1Fsha^/J; Stock No.032411) mice were obtained from the Jackson Laboratory (Bar Harbor, ME, USA). The GFAP-Cre mice were kindly provided by Michael V Sofroniew (University of California Los Angeles). We previously produced the *App*^*NL-G-F/NL-G-F*^-KI (*App*^*NL-G-F*^) mouse strain using genomic DNA of introns 15 to 17 of mouse *APP,* which humanized the Aβ sequence, and introduced KM670/671NL (Swedish), I716F (Iberian), and E693G (Arctic) mutations^41^. The WT C57BL/6 mice were purchased from Jackson Laboratory. All strains were maintained on a C57BL/6 background. The details of mouse strains used in each experiment are described in Table S3.

### Human samples

Human AD brain samples were kindly provided by Dr. John Trojanowski (University of Pennsylvania). Control samples were obtained from BioChain (San Francisco, CA, USA). Details of these samples were described previously^66^.

### Establishment of Iba1-Cre transgenic mice

The Iba1-Cre transgenic mice were generated with a standard transgenic method. The 3.5 kb 5’-flanking region of Iba1 gene was PCR-amplified from mouse genomic DNA and inserted into pBstN-NCre vector^67^ to generate Iba1-Cre plasmid. Iba1-Cre transgene was excised, gel-purified, and injected into the pronucleus of fertilized eggs of C57BL/6 J mice. The manipulated eggs were culture to the two-cell stage and transferred into oviducts of pseudopregnant foster females (ICR strain). Integration of the transgene was screened by PCR of tail DNA. Cre protein was expressed in almost all Iba1-positive microglia (Fig. S6).

### Administration of PLX3397 to mice

For pharmacological ablation of microglia, PLX3397 was administrated to mice by supplementation (290 mg/kg of PLX3397 (#C-1271; Chemgood, Glen Allen, VA, USA) of standard chow (#D10001; Research Diet, New Brunswick, NJ, USA)). 3-month-old female mice (WT or GCLC-KO) were fed with PLX3397 formulated-chow or control chow for 2 months, and brain samples were collected immediately after the end of the feeding period.

### Tissue fixation and preparation of paraffin-embedded, frozen, and vibratome-cut sections

Brain hemispheres were fixed by immersion in 4% paraformaldehyde in phosphate buffer solution (Nacalai tesque, Kyoto, Japan). 4% paraformaldehyde-fixed brains were embedded in paraffin, and 4 µm-thick sections were mounted onto MAS-GP-coated glass slides (Matsunami-glass, Osaka, Japan). For frozen sections, 4% paraformaldehyde-fixed brains were mildly shaken in 20% sucrose solution for 6 h and then 30% sucrose solution overnight at 4 °C. The fixed brains were frozen in ice-cold isopentane with Tissue Tech O.T.C. Compound (Sakura-finetek, Tokyo, Japan), and 15 µm-thick sections were mounted onto MAS-GP-coated glass slides. For vibratome-cut sections, 4% paraformaldehyde-fixed brains were cut into 50 µm-thick sections by a vibratome. Sections were stained with hematoxylin and eosin (H&E), FluoroJade C, or immunostained with antibodies.

### Histochemistry

H&E staining was carried out according to the following method. After deparaffinization, sections were stained with Mayer's hematoxylin solution (Wako, Tokyo, Japan) for 10 min, and then stained with eosin alcohol solution (Wako) for 4 min. Finally, the sections were dehydrated and coverslipped using NEW M.X (Matsunami-glass). FluoroJade C (#3,319,822; Merck Millipore, Burlington, MA, USA) staining was carried out according to the manufacturer’s instructions. Briefly, frozen sections were treated with basic ethanol (80% ethanol/1% NaOH) for 5 min, and washed in 70% EtOH and ultrapure water for 2 min, respectively. The sections were then incubated in 0.06% potassium permanganate. After washing in ultrapure water for 2 min, the sections were treated with the staining solution (Fluoro-Jade C and 2 µg/mL Hoechst diluted in 0.1% acetic acid). After washing in ultrapure water, the sections were heated at 60 °C, air dried, and incubated in xylene for 5 min. Finally, the sections were coverslipped using NEW M.X (Matsunami-glass). Floro-Styryl-Benzene (FSB) (Dojindo, Tokyo, Japan) staining was carried out according to the manufacturer's instructions. The sections were then incubated with FSB solution, and then soaked in lithium carbonate solution. After washing with 50% EtOH, the sections were coverslipped.

### Immunohistochemistry

In experiments in which we used anti-Iba1, anti-GFAP, N1D^68^ (anti- Aβ), or anti-C3, the signals were visualized with fluorescent secondary antibodies. When staining was performed with anti-GCLC, anti-cleaved caspase3, anti-TSPO (PBR), anti-PSD95 or anti-GSDME antibodies, we applied a fluorescence-indirect tyramide signal amplification (TSA) technique (TSA System; Akoya Biosciences, Marlboro, MA, USA). For anti-C1q or anti-NeuN antibody staining, a DAKO EnVision + System (Agilent Technologies, Santa Clara, CA, USA) was used as the second antibody and the signal was detected using tyramide-enhanced fluorescein isothiocyanate (FITC), as for the TSA method. Details of primary antibodies and the reaction conditions used are described in Table S1. After deparaffinization, sections were heated in an autoclave at 121 °C for 5 min in 10 mM sodium citrate buffer (pH 6.0) or reacted with protease solution (Nichirei Biosciences, Tokyo, Japan) for epitope retrieval, after which endogenous peroxidase was inactivated by 0.3% hydrogen peroxide in methanol. To block nonspecific immunoreactivity, sections were treated with the blocking solutions (0.2% Casein in PBS for the fluorescent secondary antibody method, TSA Biotin System kit for the TSA method). Primary antibodies (Table S1) diluted in TN buffer (0.1 M Tris–HCl, 0.15 M NaCl, pH 7.5) were reacted overnight at 4 °C. The sections were then washed three times in TNT buffer (0.1 M Tris–HCl, 0.15 M NaCl, 0.05% Tween20, pH 7.5) for 5 min, and treated with secondary antibodies. When the TSA method was used, the sections were treated with biotinylated goat anti-mouse/rabbit IgG (1:1000 dilution, Vector Laboratories, Burlingame, CA) for 1 h, and then incubated with HRP-conjugated-avidin for 30 min (1:100 dilution in TN buffer, TSA System) at room temperature. Visualization of stained cells was achieved with tyramide-enhanced FITC or rhodamine (1:50 dilution in amplification solution; supplied in the TSA System) for 10 min. When the fluorescent secondary antibody method was used, the sections were treated with Alexa 488- or Alexa 555-conjugated anti-mouse/rabbit IgG (1:500 dilution, Molecular Probes, Eugene, OR). Finally, the sections were coverslipped using ProLong Gold Antifade Reagent (Thermo Fisher Scientific, Waltham, MA, USA). For C3 antibody staining we used vibratome-cut sections. After blocking with blocking solution (0.2% casein, 0.3% Triton X-100, and 3% goat serum diluted in PBS) for 2 h at room temperature, anti-C3 (1:100 dilution) antibody-stained sections were reacted overnight at 4 °C. The sections were then washed with PBS and treated with secondary antibody (diluted in PBS containing 0.3% Triton X-100) for 2 h at room temperature. After washing with PBS, the sections were coverslipped. The immunostained sections were scanned on a NanoZoomer NDP system (Hamamatsu Photonics, Shizuoka, Japan) with 20 × resolution, or on a FV3000 confocal microscope (Olympus, Tokyo, Japan) with 4, 10 or 20 × resolution. The signals were quantified using Metamorph Imaging Software (Molecular Devices, San Jose, CA, USA). The fluorescence intensity of each protein was calculated as the product of the average fluorescence intensity and fluorescence area.

### Western blotting

Frozen cortex or hippocampal samples were homogenized in 50 mM Tris–HCl (pH 7.5), 150 mM NaCl containing 1% Triton X-100, protease inhibitor cocktail and phosphatase inhibitor cocktail. After incubation on ice for 1 h, the homogenates were centrifuged at 20,400×*g* for 20 min at 4 °C, and the resulting supernatants were appropriately diluted and used in the assays. For the western blotting of HMGB1, frozen cortices were homogenized in 50 mM Tris–HCl (pH 7.5) containing protease inhibitor cocktail and phosphatase inhibitor cocktail. After ultracentrifugation at 200,000×*g* for 20 min at 4 °C the resulting supernatants were used in the assays. Protein concentrations were determined using a BCA protein assay kit (Pierce, Rockford, IL, USA). An equivalent amount of protein from each animal was mixed with 4 × sample buffer with or without 2-mercaptoethanol, then separated by SDS–polyacrylamide gel electrophoresis, and transferred electrophoretically to a PVDF membrane (Merck Millipore). The membrane was treated with ECL prime blocking solutions (GE Healthcare, Little Chalfont, UK) and reacted overnight at 4 °C with each antibody (Table S1) diluted in blocking buffer. The membrane was washed three times in Tris-buffered saline with Tween 20 (TBS-T) for 10 min, and treated with HRP-conjugated anti-rabbit or anti-mouse IgG (GE Healthcare) for 1 h. Immunoreactive bands on the membrane were visualized with ECL select (GE Healthcare) and scanned with a LAS-4000mini LuminoImage analyzer (Fuji Film, Tokyo, Japan). Details of primary antibodies are provided in Table S1.

### Quantitative real-time PCR (qRT-PCR) analysis

Total RNA was isolated from cortical samples by using RNAiso plus (Takara, Shiga, Japan) according to the manufacturer’s instructions. Briefly, tissues were homogenized in 500 µL of RNAiso plus, with total RNA separated by mixing with 100 µL of chloroform, and isolated by isopropanol precipitation. Removal of genomic DNA and reverse transcription were carried out with ReverTra Ace qPCR RT Kit (Toyobo, Osaka, Japan) according to the manufacturer's instructions. Real-time qPCR was performed using an Applied Biosystems QuantStudio 12 K Flex real time PCR system (Thermo Fisher Scientific, San Jose, CA, USA) with THUNDERBIRD SYBR qPCR Mix (Toyobo), 6 pmol of primers, and aliquots of cDNA. Details of the PCR primers used are provided in Table S2. The PCR conditions used were as follows: 50 °C for 2 min, 95 °C for 1 min; 40 cycles of 95 °C for 10 s, and 60 °C for 30 s. All qRT-PCR were run in technical duplicates. The standard curve method based on the reference value was used to calculate relative level of each gene. Finally, expression levels of target genes were determined by the ratio between the levels of target genes and the levels a housekeeping gene (*gapdh*).

### Magnetic resonance imaging

We sequentially conducted magnetic resonance imaging (MRI) of the brains of WT, GCLC-KO, PLX-administrated GCLC-KO, and GSDME-KO X GCLC-KO mice. Mice were anesthetized with 1.5% (v/v) isoflurane and anchored in the apparatus. During the scanning, the depth of anesthesia was monitored with a breathing sensor. Coronal T2-weighted (T2W) MRI scans (2D TurboRAGE) of the whole brain were performed with a vertical-bore 9.4 T Bruker AVANCE 400WB imaging spectrometer with a 250 mTm^−1^ actively shielded imaging gradient insert (Bruker BioSpin, Billerica, MA) controlled by Paravision software. T2W scans were performed with the following parameter settings: TR (repetition time) = 4342.2 ms, TE (echo time) = 53.8 ms, matrix dimensions = 256 × 256, flip angle = 180 degrees, field of view = 1.8 cm × 1.8 cm. We used a slice thickness of 0.5 mm and 29 slices with a scan time of 15 min to image the whole brain. Within the 29 scanned images, images containing the hippocampal area were selected for further analysis. The hippocampal volume and cortex thickness of each mouse were calculated using ImageJ software.

### Quantification of glutathione concentration

Assays were performed on brain tissue and plasma samples using the GSSG/GSH Quantification Kit (Dojindo, Kumamoto, Japan*).* Frozen cortical samples (the weights of which were determined beforehand) were homogenized in 5% 5-sulfosalicylic acid (SSA), and the mixtures were centrifuged at 8000×*g* for 10 min at 4 °C. The resulting supernatants were diluted in a 10 times volume, and the dilutions were used in the assay. Blood was collected from the right ventricles of mice, and an equivalent volume of 5% SSA was immediately added to the blood sample. The mixtures were centrifuged at 8000×*g* for 10 min at 4 °C. The resulting supernatants were diluted in a 5 times volume, and the dilutions were used in the assay. The assay was performed according to the manufacture’s instruction and calculated the reduced and oxidized glutathione content in fixed quantities of brain tissue or blood.

### LC–MS/MS and pathway analysis

LC–MS/MS samples were prepared according to a Filter-aided Sample Preparation (FASP) method^69^. Briefly, the frozen cortical samples were homogenized in SDT-lysis buffer (100 mM Tris–HCl (pH 7.6) containing 0.1 M DTT) using an ultrasonic homogenizer, and the homogenates were heated at 95 °C for 3 min. After centrifugation at 16,000×*g* for 5 min, the resulting supernatant was used for processing. The protein sample (250 µg protein /30 µL) was mixed with 270 µL of UA (100 mM Tris–HCl (pH 8.5) containing 8 M Urea) in the filter unit, and centrifuged at 14,000×*g* for 15 min. The filter was washed two times with 100 μL of UA, centrifuged at 14,000×*g* for 15 min, and incubated with 100 μL of IAA solution (0.05 M iodoacetamide in UA) for 20 min in the dark. After washing twice with UA as above, 100 μL of 50 mM AmBic (50 mM NH_4_HCO_3_ in water) was added to the filter unit and the filter was centrifuged at 14,000×*g* for 10 min. The flow-through was discarded, and then the filter unit was incubated with 40 μL of 50 mM AmBic with trypsin (1/100 concentration of protein) overnight at 37 °C. After incubation, 40 μL of 50 mM AmBic was added to the filter unit, and the flow-through resulting from centrifugation at 14,000×*g* for 10 min was collected. The collected protein samples were used for LC–MS/MS analyses. LC–MS/MS analysis was performed using an Advance nanoLC (BrukerMichrom, Auburn, CA, USA) and LTQ linear ion trap mass spectrometer (Thermo Fisher Scientific) equipped with a NANOHPLC capillary column C18 (0.075 mm ID × 150 mm length, 3 µm particle size, Nikkyo Technos, Tokyo, Japan) using a linear gradient (25 min, 5–35% CH_3_CN /0.1% formic acid) at a flow rate of 300 nL/min. The resulting MS and MS/MS data were searched against the Swiss-Prot database using MASCOT software (Matrix Science, London, UK).

### Cytokine array

The cytokine assay was performed using a Proteome Profiler Mouse Cytokine Array Kit (R and D systems, Minneapolis, MN, USA*).* Frozen cortical samples were homogenized in 50 mM Tris–HCl (pH 7.5), 150 mM NaCl containing 1% Triton X-100, protease inhibitor cocktail and phosphatase inhibitor cocktail. The homogenates were centrifuged at 20,400 × g for 20 min at 4 °C, and the resulting supernatants were appropriately diluted and used in the assays, which were conducted according to the manufacturer’s instructions.

### Statistical analyses

All analyses were completed with GraphPad Prism7 Software (San Diego, CA, USA). Differences between groups were examined for statistical significance by Student’s t-test unless otherwise stated. Some data were analyzed by two-way ANOVA.

### Ethical statement

Research with human subjects were conducted in compliance to guidelines: “Ethical Regulations for Research Involving Human Subjects” and “Supplementary Ethical Regulations for Research Involving Human Subjects”. Experiments with human samples were approved by the Research Ethics Committee, the Wako Safety Center in RIKEN (approval number: Wako3 30-4(2)). All animal experiments were conducted in compliance to guidelines: “Regulations for the Animal Experiments” and “Wako Institute Animal Experiment Handbook”. Animal experiments were approved by the Wako Animal Experiment Committee in RIKEN (approval number: W2021-2-020(1)). All animal experiments were performed in accordance with the ARRIVE guidelines. All genetic recombinant experiments were conducted in compliance to guidelines: “Genetic Recombinant Experiment Safety Control Regulations” and “Supplementary Regulations for Genetic Recombinant Experiment Safety Control”. Genetic recombinant experiments were reviewed by Safety Officer in RIKEN, and approved by the director of RIKEN Wako Campus (approval number: 2022-029(1)). All methods were carried out in accordance with concerned laws and Biosafety Manual in RIKEN.

## Supplementary Information

Supplementary Information.

Supplementary Figures.

Supplementary Tables.

## Data Availability

The datasets are available from the corresponding authors upon reasonable request. The Iba1-Cre transgenic mice are available from Yoshihiro Yoshihara under a material transfer agreement. Other genetically modified mice are available as described in the Materials and Method section.

## Abbreviations

GCLC: Glutamate cysteine ligase catalytic subunit
GCLM: Glutamate cysteine ligase modifier subunit
GSH: Reduced glutathione
GSSG: Oxidized glutathione
ROS: Reactive oxygen species
C1q: Complement component 1q
C3: Complement component 3
GSDMD: Gasdermin D
GSDME: Gasdermin E
DAM: Disease associated microglia
DAA: Disease associated astrocyte
Iba1: Ionized calcium-binding adapter molecule 1
GFAP: Glial fibrillary acidic protein
NeuN: Neuronal nuclei
HO1: Hemeoxygenase 1
CCL: CC Chemokine ligand
CXCL: C-X-C motif ligand
AD: Alzheimer’s disease
App: Amyloid precursor protein
Aβ: Amyloid beta
*App*^*NL-G-F*^: *App*^*NL-G-F*^ knockin mouse
