# Supplementary material for: Neuronal glutathione loss leads to neurodegeneration involving gasdermin activation
Source: Sci Rep. 2023 Jan 20;13:1109. doi: 10.1038/s41598-023-27653-w (PMC9859798; doi:10.1038/s41598-023-27653-w)
Supplement: Supplementary file 2 — Supplementary Figures. [file 41598_2023_27653_MOESM2_ESM.pdf]

Figure S1

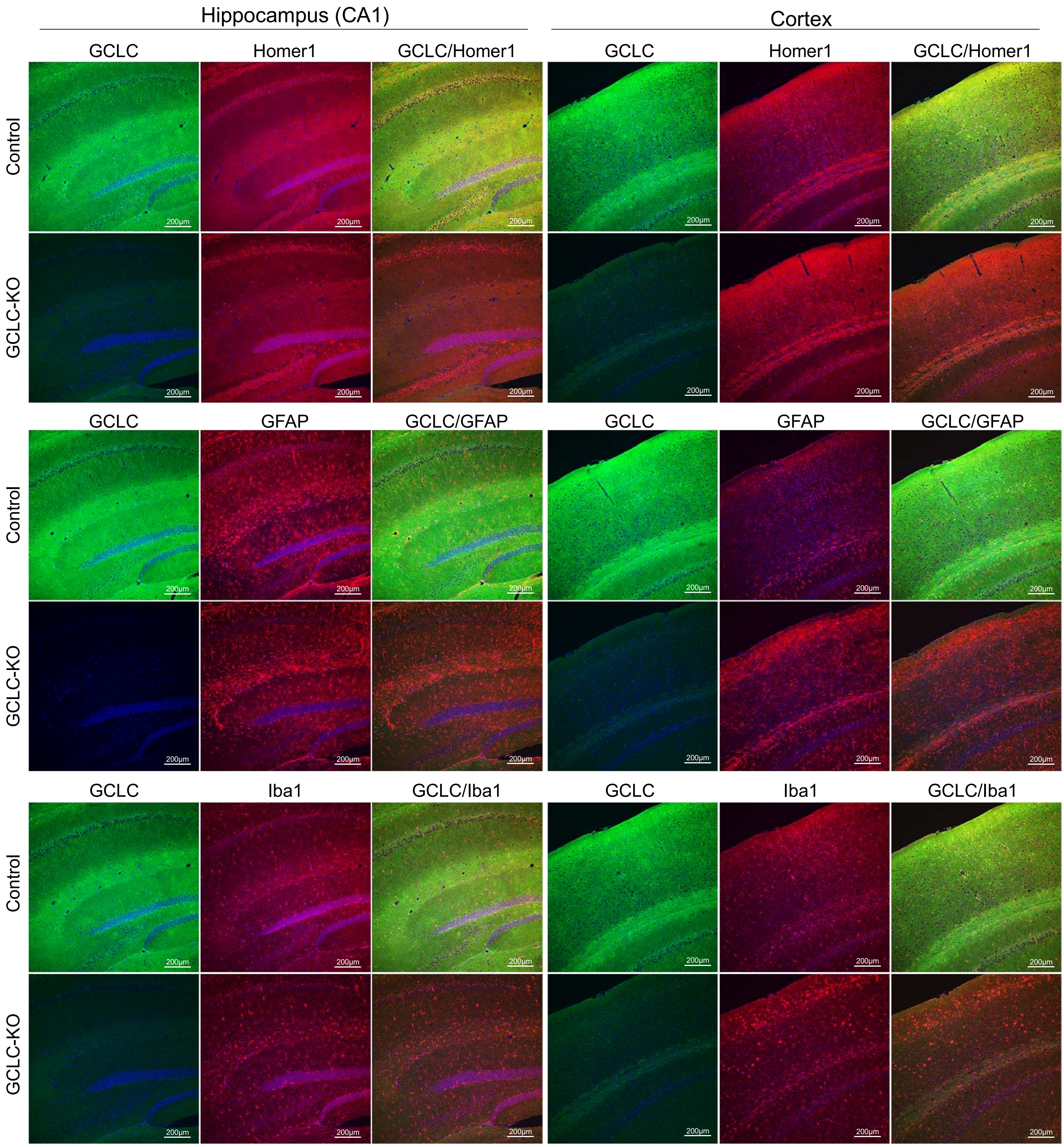

Figure S1 GCLC expression in GCLC^{floxed} X CamKII-Cre mice

Brain sections of 3-month-old GCLC^{floxed} X CamKII-Cre (GCLC-KO) and control mice were immunostained with GCLC and Homer1(neuronal cell marker)(upper panels)/GFAP (middle panels)/Iba1 (lower panels) antibodies. The majority of GCLC were expressed in neuronal cells. GCLC was deleted in Homer1 expressing-neuronal cells of GCLC-KO mouse.

Figure S2

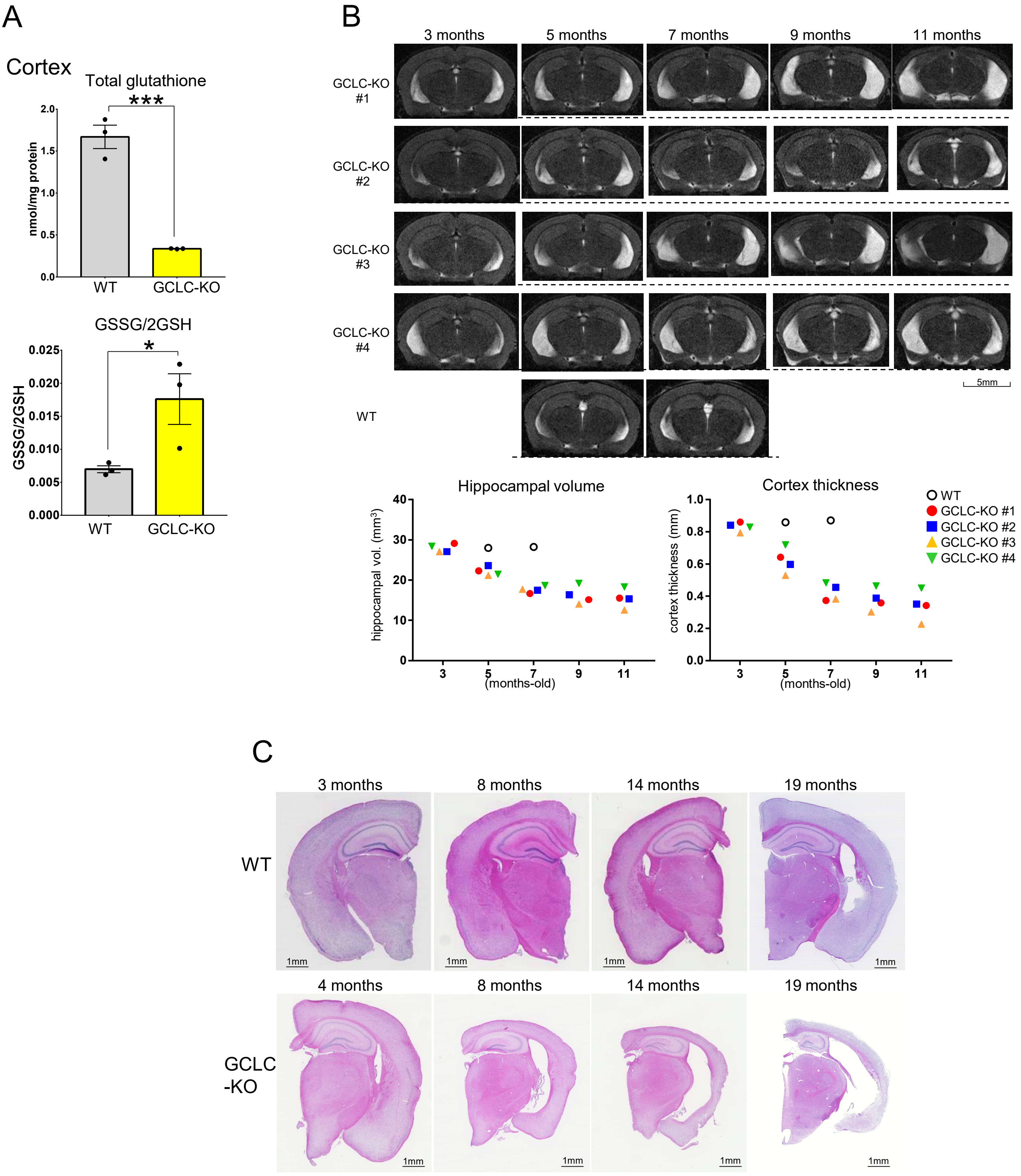

Figure S2 Characterization of GCLC^{floxed} X CamKII-Cre (GCLC-KO) mice
(A) Total glutathione levels (left) and GSSG (oxidized form) to GSH ratio (right) in the cortices 6-month-old WT and GCLC-KO mice were determined. Values shown in the graph represent nmol/mg protein (total levels) or ratio of GSSG/2GSH (GSSG ratio) expressed as the mean level ± SEM (n=3; *p<0.05 , **p<0.01, ***:p<0.001) .(B) MRI scans of GCLC-KO mouse brains (n=4) were performed every two months from 3 to 11 months of age. Representative images and quantitative data are shown in Fig.1B and C. MRI scans of a WT mouse (n=1) were also performed at 5 and 7 months of age. Hippocampal volume and cortical thickness of individuals were shown in the plots. WT mouse showed no brain atrophy.(C) Brain sections from 3-, 8-, 14-, and 19-month-old WT mice were stained by H&E.

Figure S3

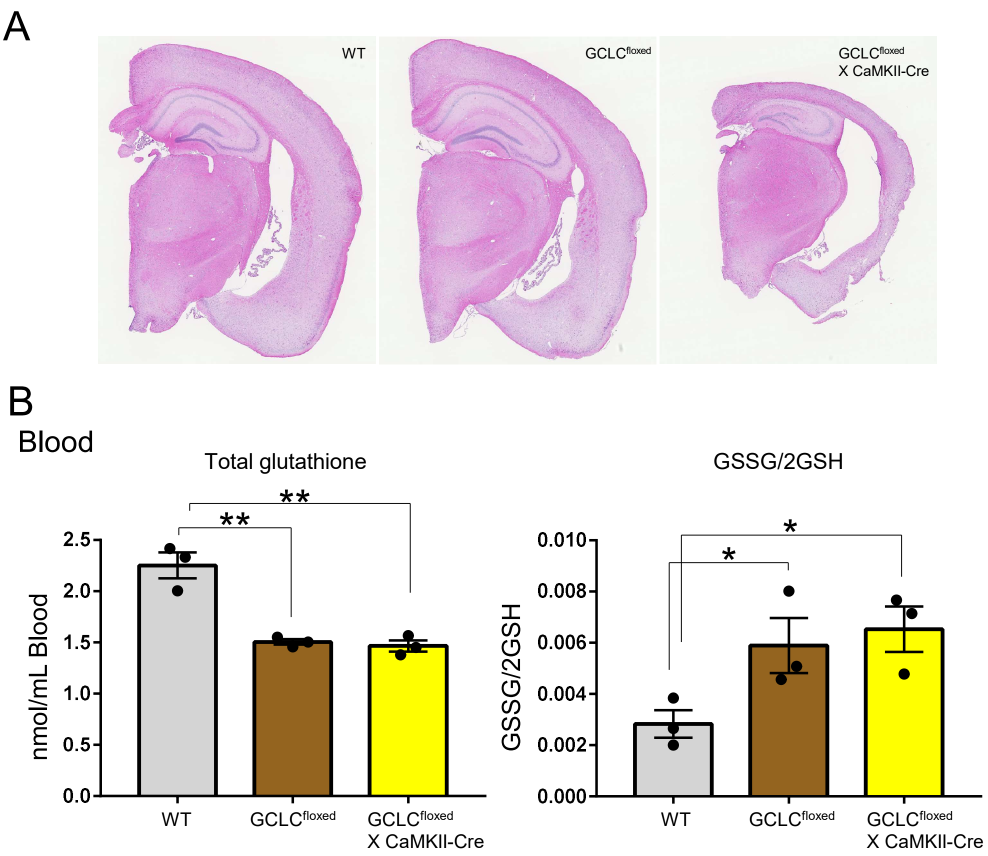

Figure S3 Characterization of GCLC^{floxed} mice

(A) Brain sections from 14- month-old WT, GCLC^{floxed} (Cre-negative), and GCLC^{floxed} X CaMKII-Cre (GCLC-KO) mice were stained by H&E. (B) Total glutathione levels (left) and GSSG (oxidized form) to GSH ratio (right) in the cortices 6-month-old WT, GCLC^{floxed}, and GCLC^{floxed} X CaMKII-Cre (GCLC-KO) mice were determined. Values shown in the graph represent nmol/mg protein (total levels) or ratio of GSSG/2GSH (GSSG ratio) expressed as the mean level ± SEM (n=3; *$p<0.05$, **$p<0.01$). GCLC-KO mice shows lowered levels of glutathione in blood probably because introduced *loxp* sequences interrupt GCLC expression. However, Cre-negative mice show no brain atrophy like GCLC^{floxed} X CaMKII-Cre.

Figure S4

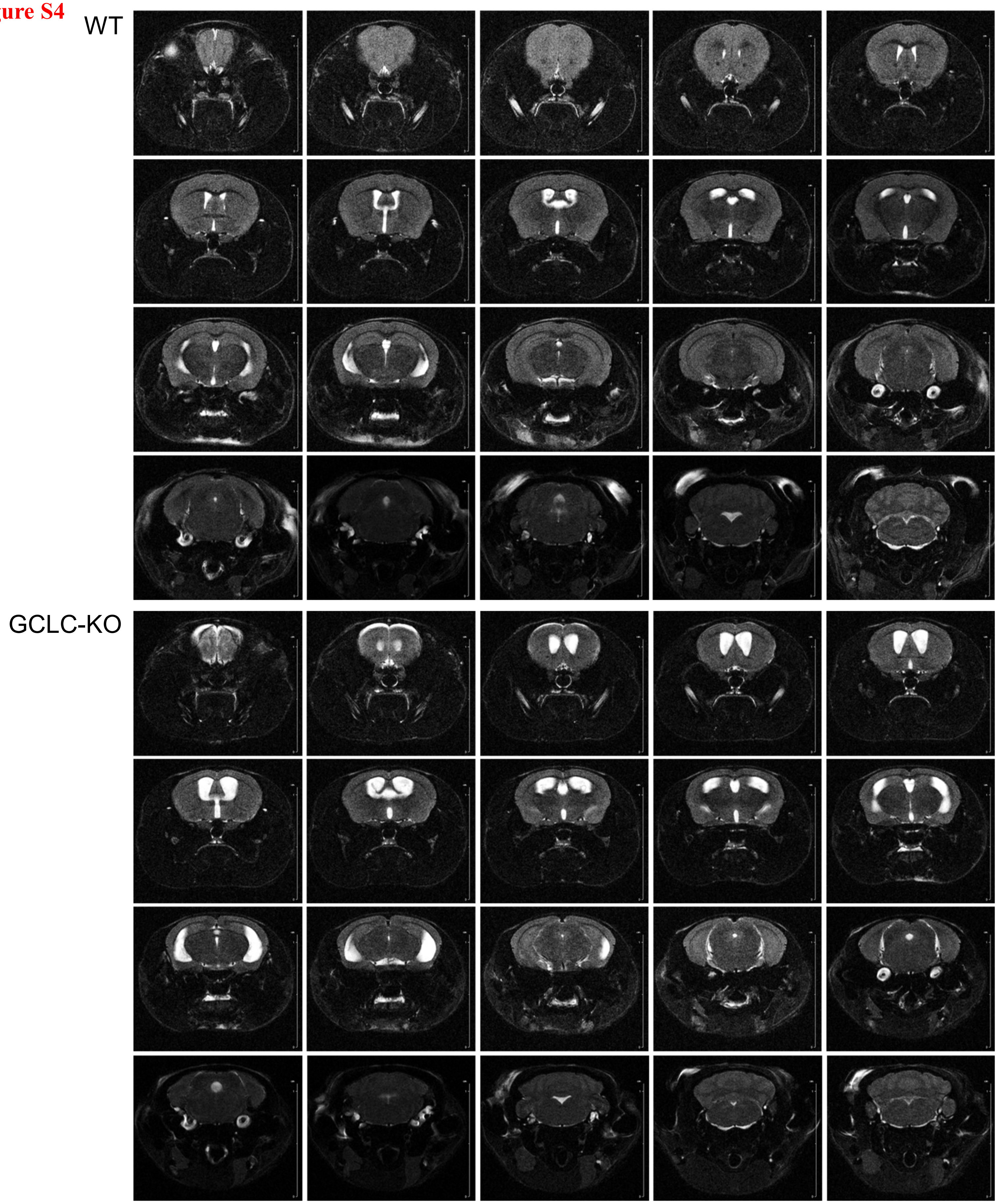

Figure S4 MRI images of 7-month-old WT and GCLC-KO mouse

We used a slice thickness of 0.5mm and 29 slices with a scan time of 22 min 47s to image the whole brain. Within the 29 scanned images, 20 images were shown in the Figure. Within the 29 scanned images, 8-10 images were containing the hippocampal. We measured the area value of hippocampus of each image using ImageJ software, and calculated the hippocampal volume with the area value and the slice thickness (0.5mm). To determine cortex thickness, the thickness of same cortical position of each mouse was measured by ImageJ software.

Figure S5

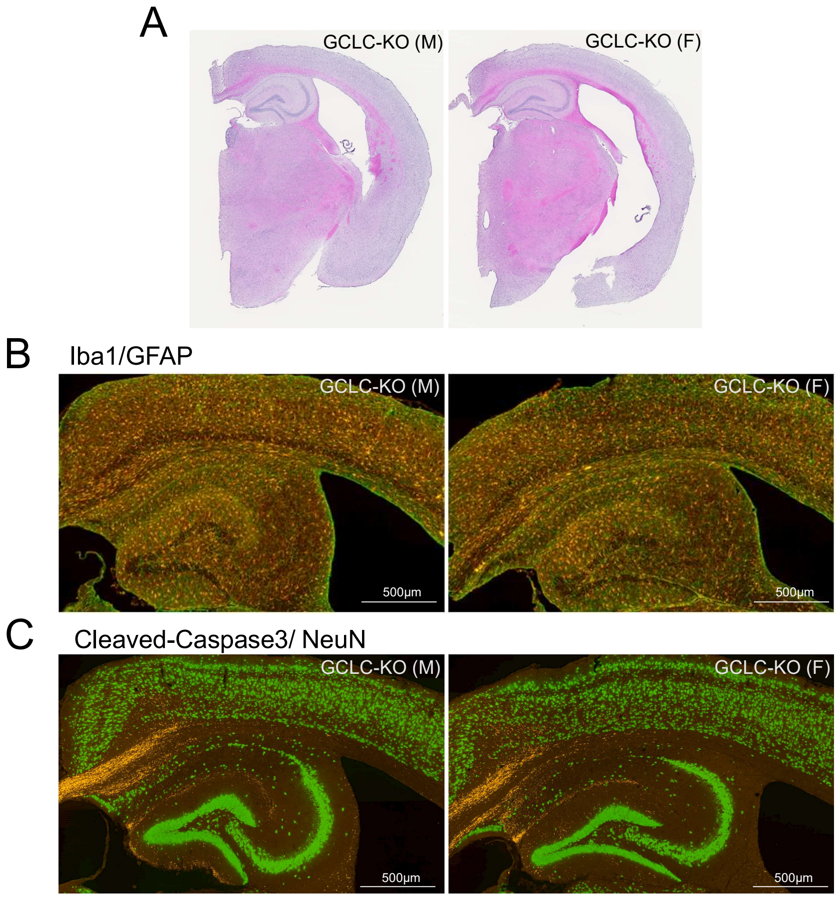

Figure S5 Characterization of male and female GCLC-KO mice
(A-C) Brain sections from 8-month-old male (M) and female (F) GCLC-KO mice were stained by H&E (A), immunostained with Iba1(Red) and GFAP (Green) antibodies (B), or immunostained with NeuN (Green) and cleaved-Caspase3 (red) antibodies (C). There is no significant difference in neuronal cell death and neuroinflammation between male and female.

Figure S6

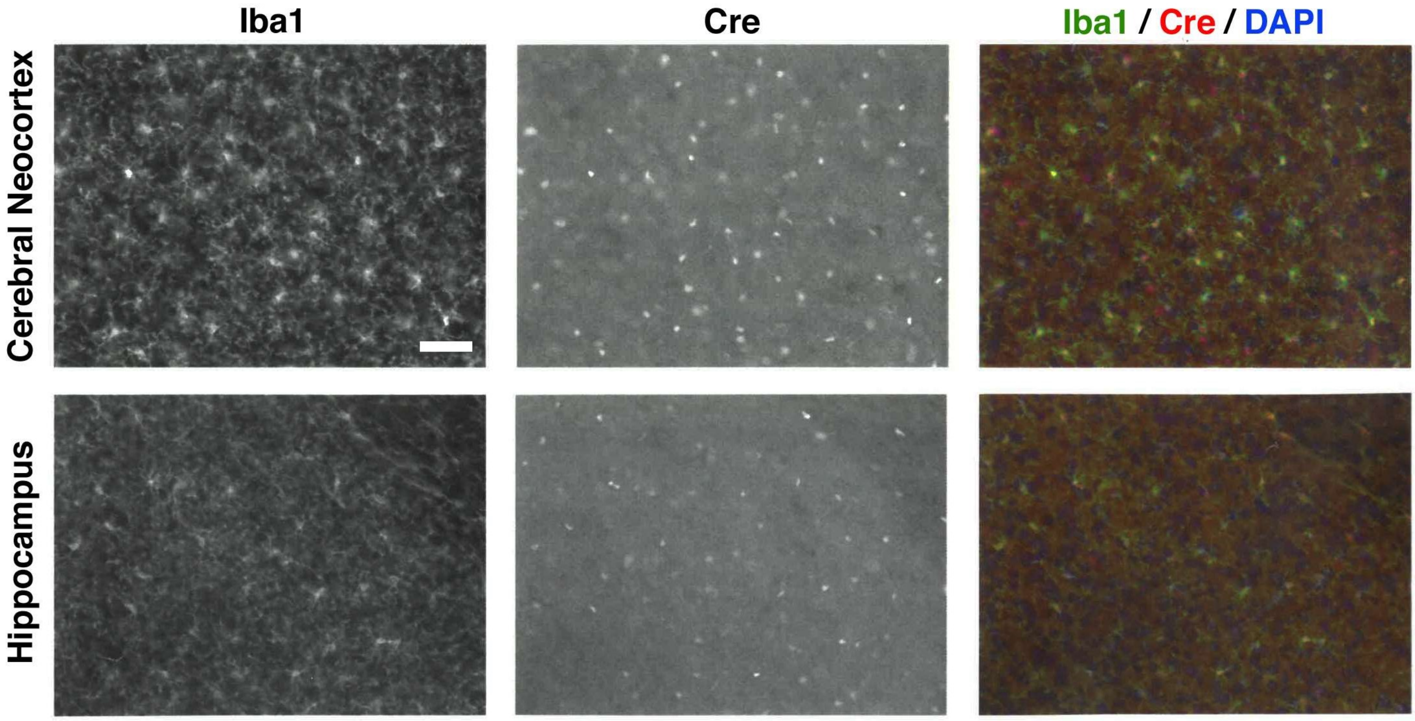

Figure S6　Characterization of Iba1-Cre mouse
Brain sections from Iba1-Cre mice were immunostained with Iba1(left) and Cre (middle) antibodies. Cre protein was expressed in almost all Iba1-positive cells.

Figure S7

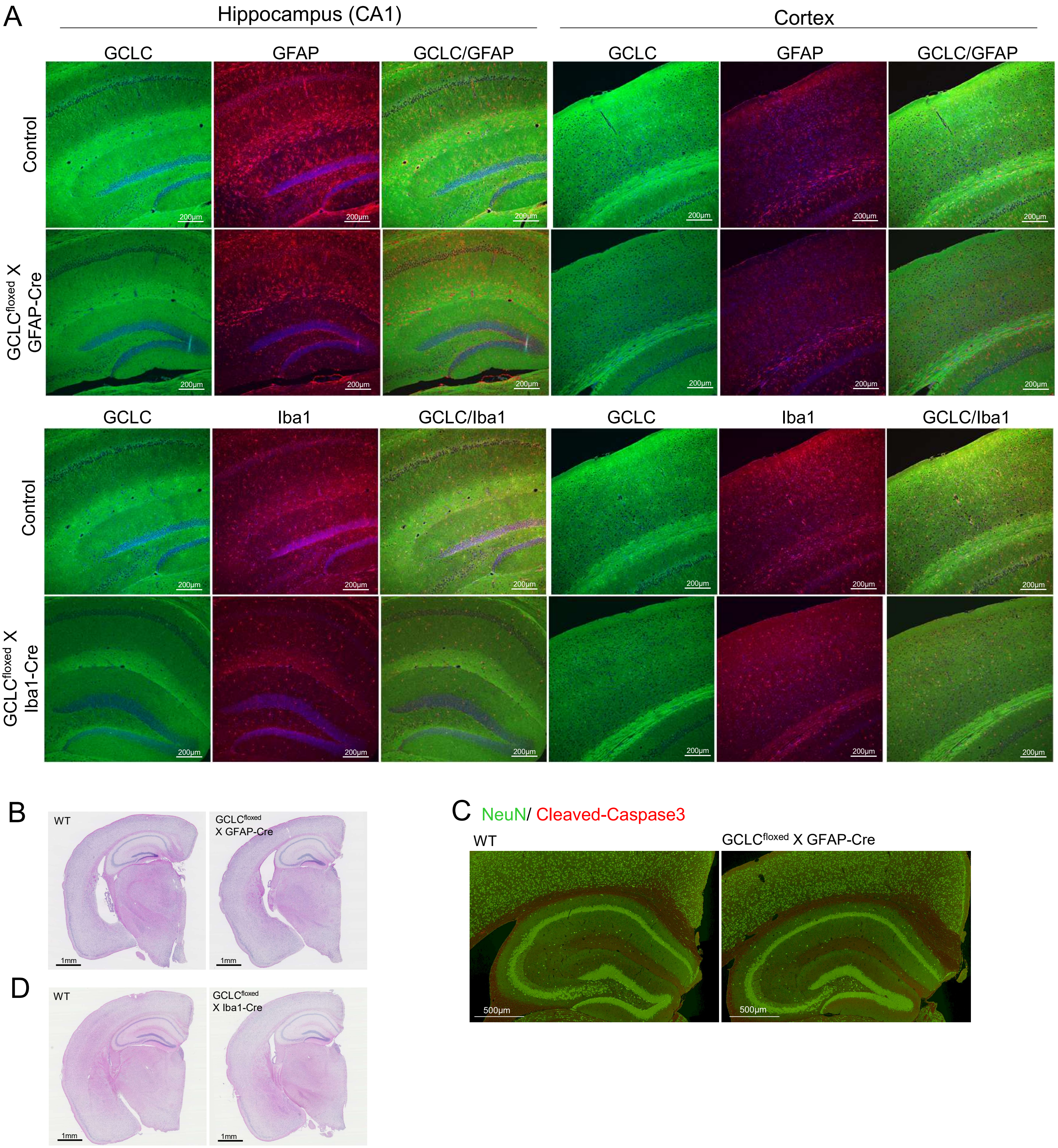

Figure S7 Characterization of GCLC^{floxed} X GFAP-Cre and GCLC^{floxed} X Iba1-Cre mice
(A) Brain sections of 3-month-old GCLC^{floxed} X GFAP-Cre or GCLC^{floxed} X Iba1-Cre mice were immunostained with GCLC and GFAP/Iba1 antibodies. (B) Brain sections of 8-month-old GCLC^{floxed} X GFAP-Cre mice were stained with H&E. (C) Brain sections of 8-month-old GCLC^{floxed} X GFAP-Cre mice were immunostained with NeuN (Green) and cleaved-Caspase3 (red). (D) Brain sections of 3-month-old GCLC^{floxed} X Iba1-Cre mice were stained with H&E.

Figure S8

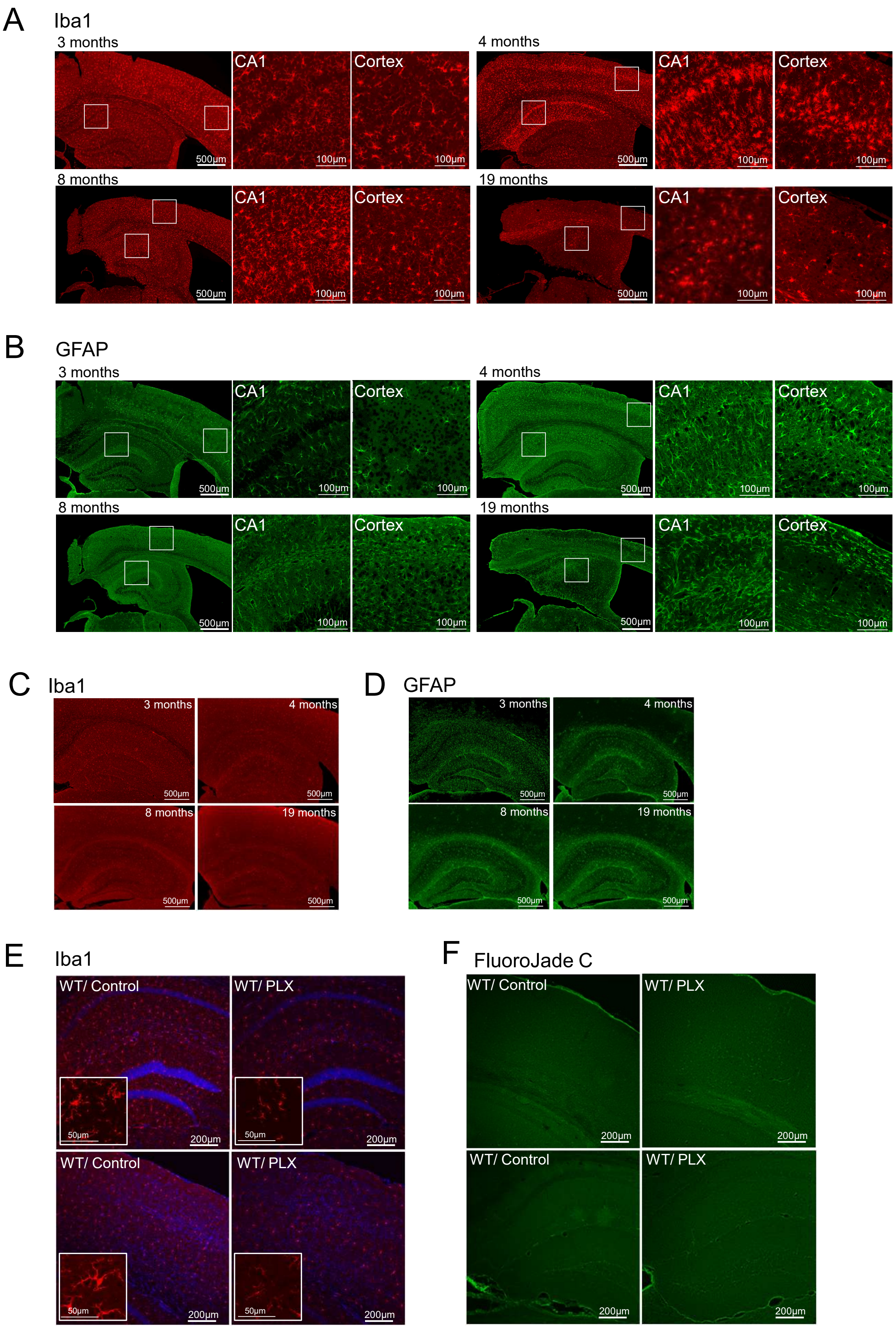

Figure S8 Neuroinflammation in WT and GCLC-KO mouse
(A) Brain sections of 3-,4-,8- and 19-month-old GCLC-KO mice were immunostained with Iba1(A) or GFAP (B) antibody. A and B are high magnification images on CA1 and cortex regarding Fig. 2B and C, respectively. (C, D) Brain sections of 3-,4-,8- and 19-month-old WT mice were immunostained with Iba1(C) or GFAP (D) antibody. (E, F) Brain sections from PLX- or control-chow treated WT mice were immunostained with Iba1 antibody (E), stained with FluoroJade C (F).

Figure S9

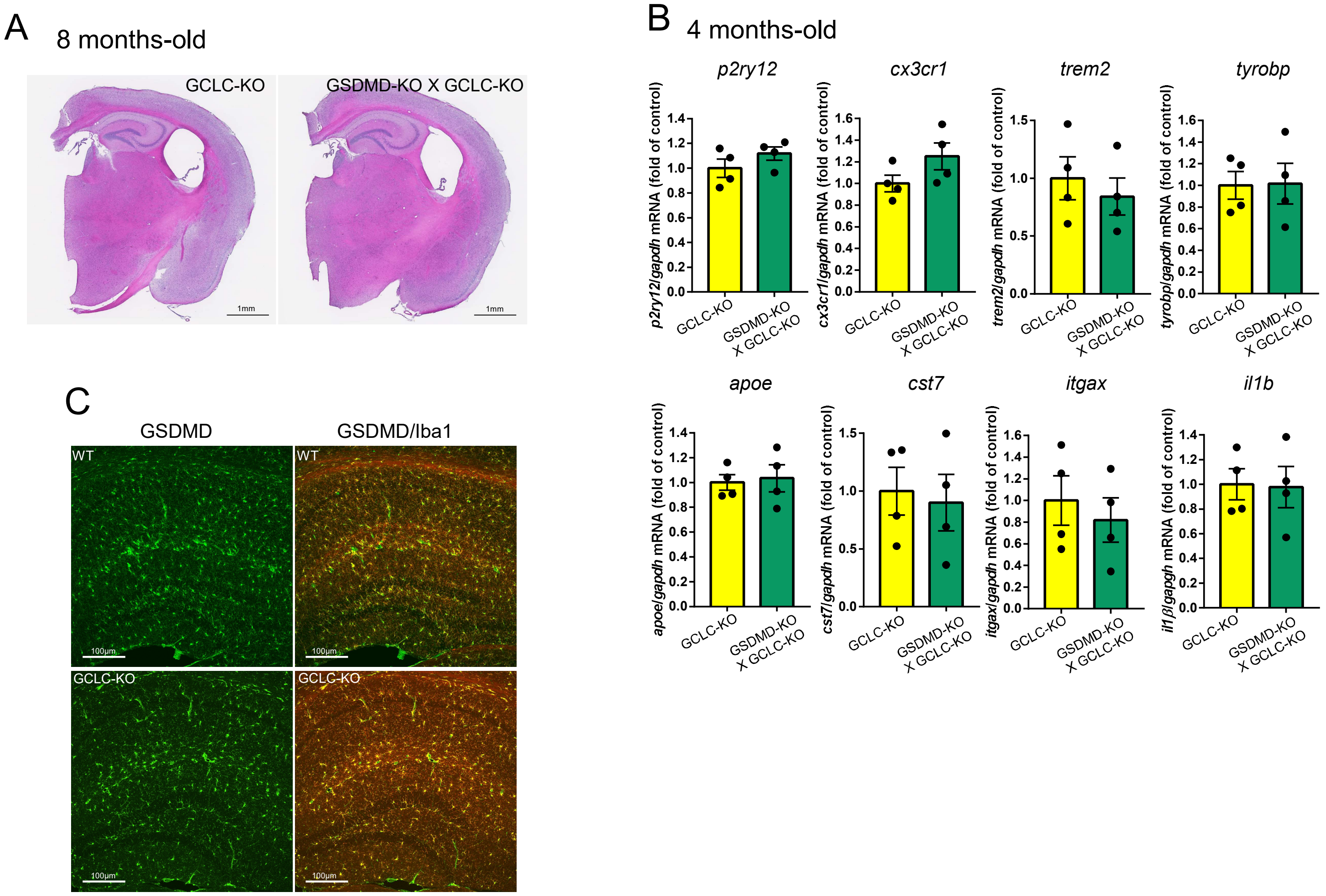

Figure S9 Characterization of GSDMD-KO X GCLC-KO mice

(A) Brain sections of 8-month-old GSDMD-KO X GCLC-KO mice were stained by H&E. (B) mRNA levels of microglial markers in GCLC-KO and GSDMD-KO X GCLC-KO were determined by qRT-PCR. Values shown in the graph represent the mean relative expression level ± SEM calculated with the standard curve method (n=3). (C) Brain sections of 3-month-old WT or GCLC-KO mice were immunostained with GSDMD and Iba1 antibodies.

Figure S10

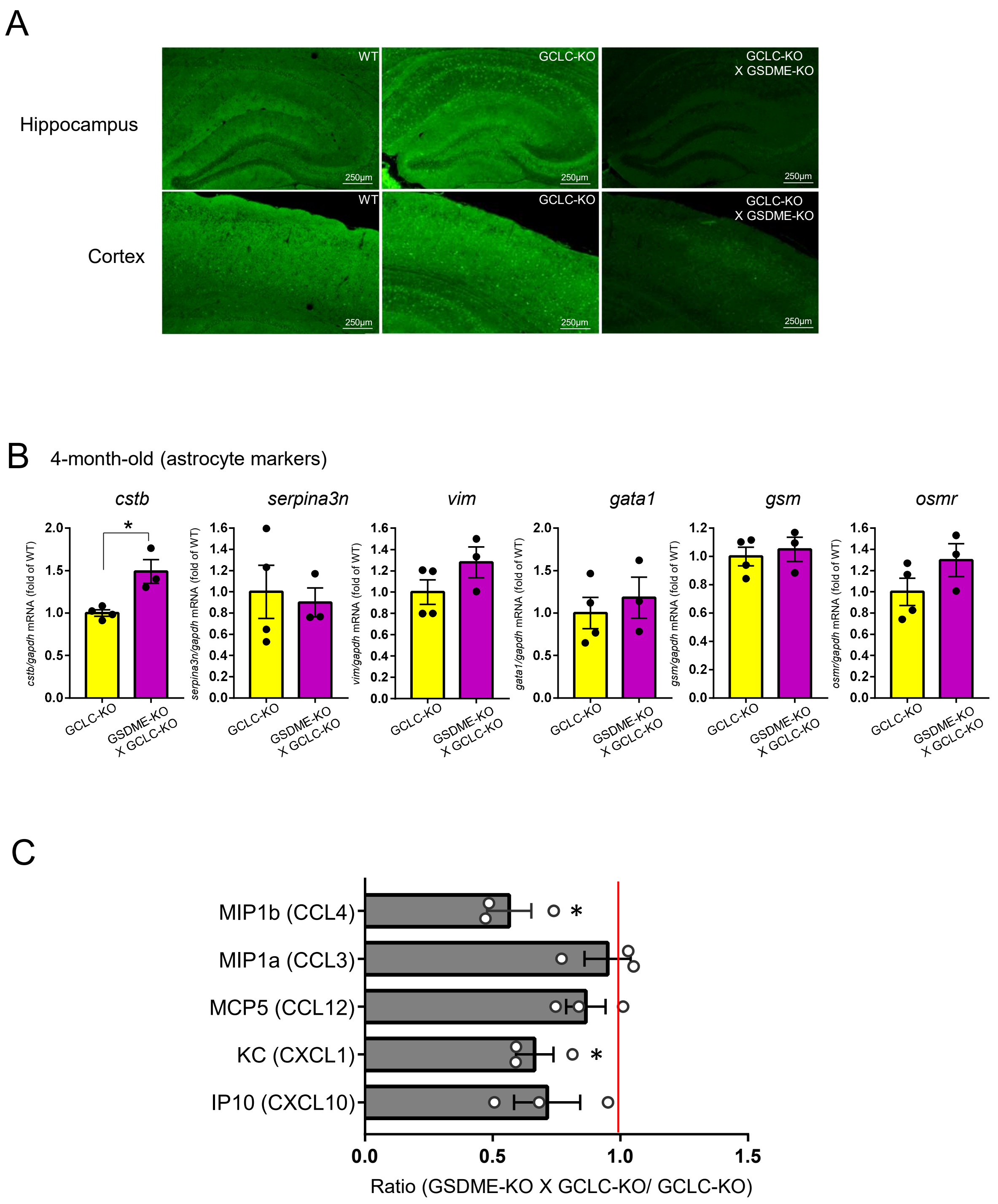

Figure S10 mRNA levels of astrocyte markers and chemokine levels in GSDME-KO X GCLC-KO
(A) Brain section of 3-month-old WT, GCLC-KO and GSDME-KO X GCLC-KO mice were immunostained with GSDME antibodies (B) mRNA levels of astrocyte markers in GSDME-KO X GCLC-KO and GCLC-KO were determined. Values shown in the graph represent the mean relative expression level ± SEM (n=3 or 4; *p<0.05). (C) Protein levels of cytokines in 8-month-old GCLC-KO and GSDME-KO X GCLC-KO were determined by cytokine array. Values shown in the graph represent the mean relative expression level of cytokines in GSDME-KO X GCLC-KO/ GCLC-KO (n=3; *p<0.05).

Figure S11

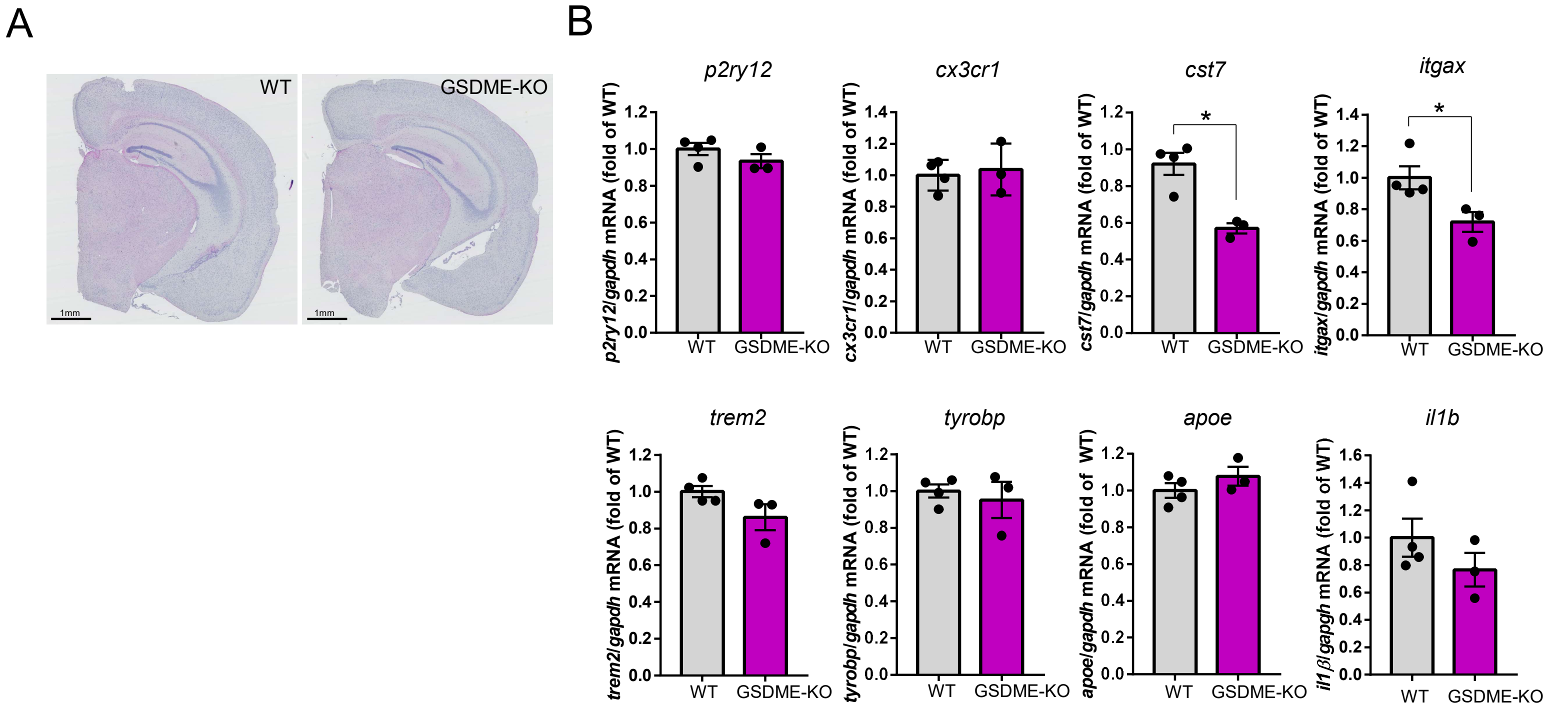

Figure S11 Characterization of GSDME-single KO mice

(A) Brain sections of 8-month-old WT and GSDME-KO mice were stained by H&E. (B) mRNA levels of microglial markers in 8-month-old WT and GSDME-KO mice were determined by qRT-PCR. Values shown in the graph represent the mean relative expression level ± SEM calculated with the standard curve method (n=4 (WT), n=3 (GSDME-KO); *p<0.05).

Figure S12

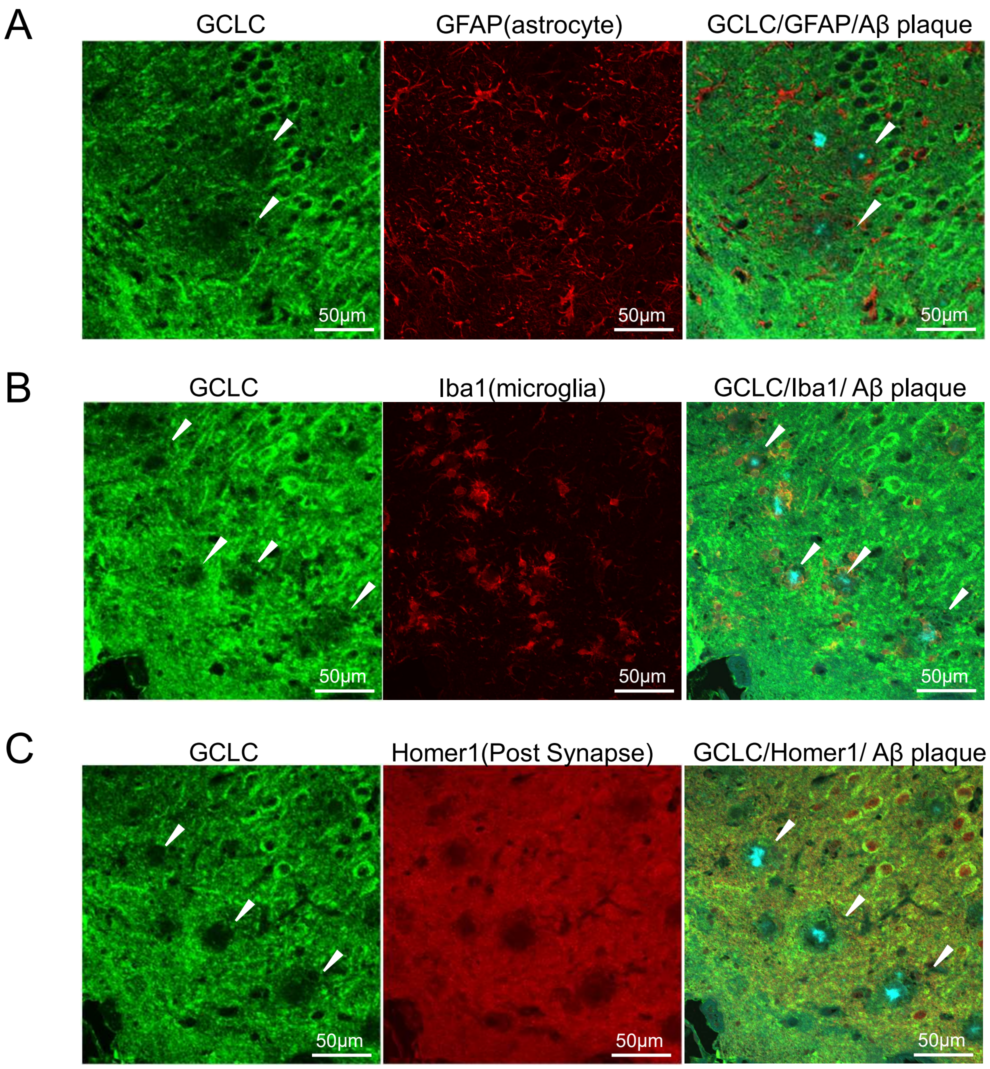

Figure S12 GCLC expression in *App*$^{NL\text{-}G\text{-}F}$ mouse brain
(A-C) Brain sections of 24-month-old *App*$^{NL\text{-}G\text{-}F}$ mice were stained with Floro-Styryl-Benzene (amyloid plaques; cyan), immunostained with anti-GCLC (green) and immunostained with anti-GFAP (A)/Iba1 (B)/Homer1(C; post synapse marker) (red) antibodies. Many GCLC signals were merged with Homer1 signals. GCLC signals were eliminated with reduction of post synaptic markers around amyloid plaques.

Figure S13

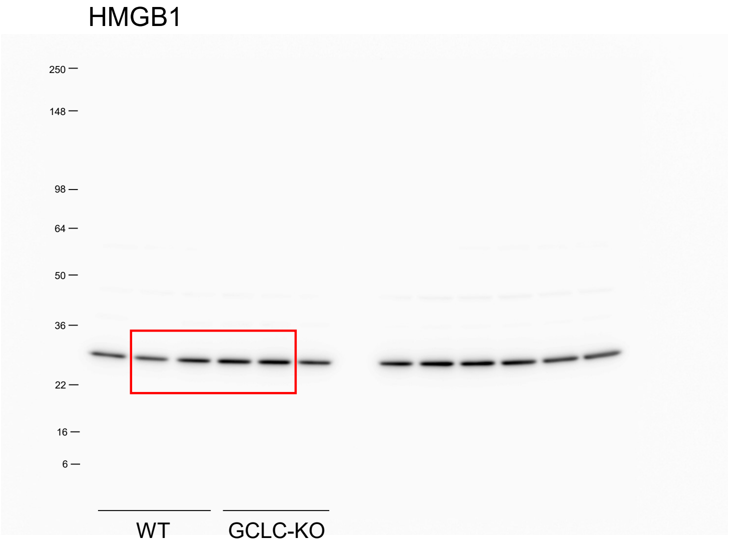

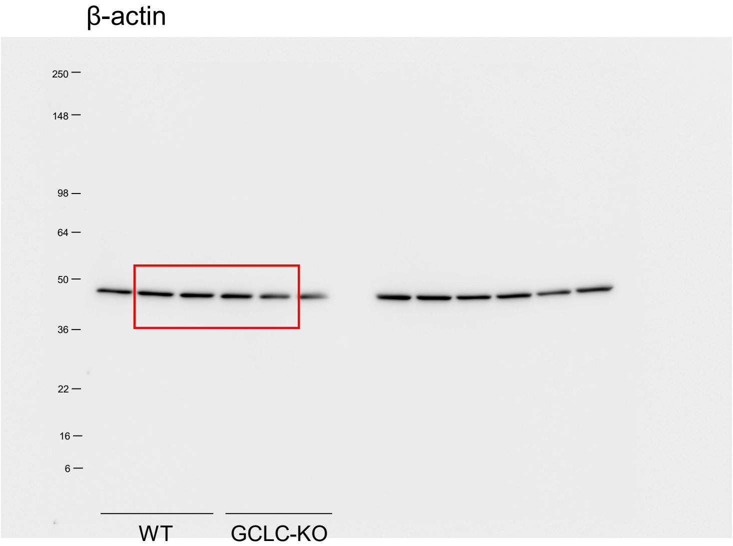

Figure S13 The full image for Figure 2G
Red frames are regions shown in Figure 2G. The images contain bands that are unrelated to this paper.

Figure S14

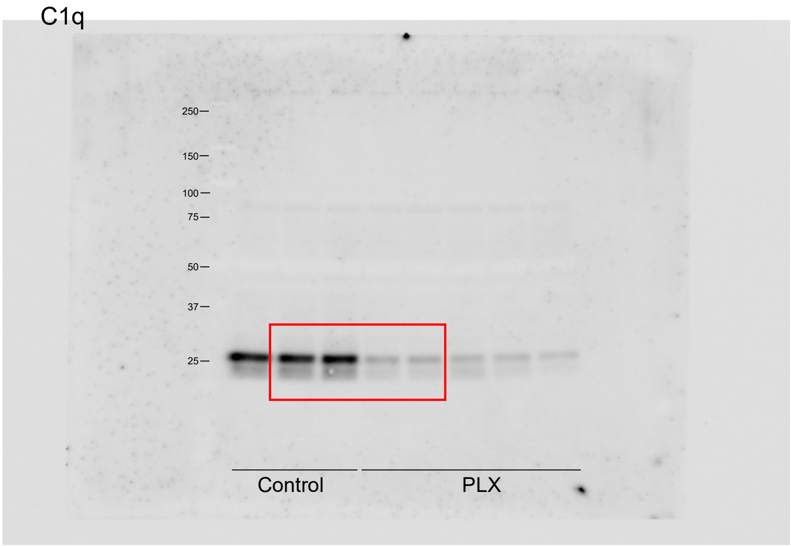

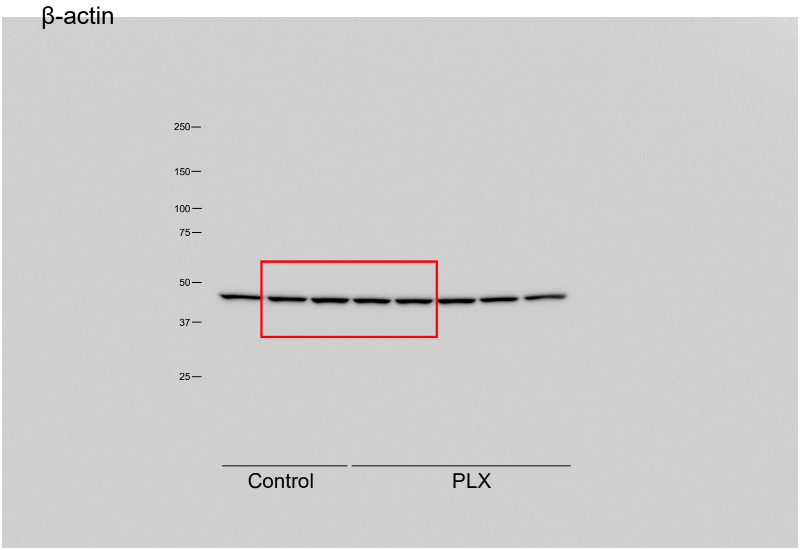

Figure S14 The full image for Figure 5F
Red frames are regions shown in Figure 5F

Figure S15

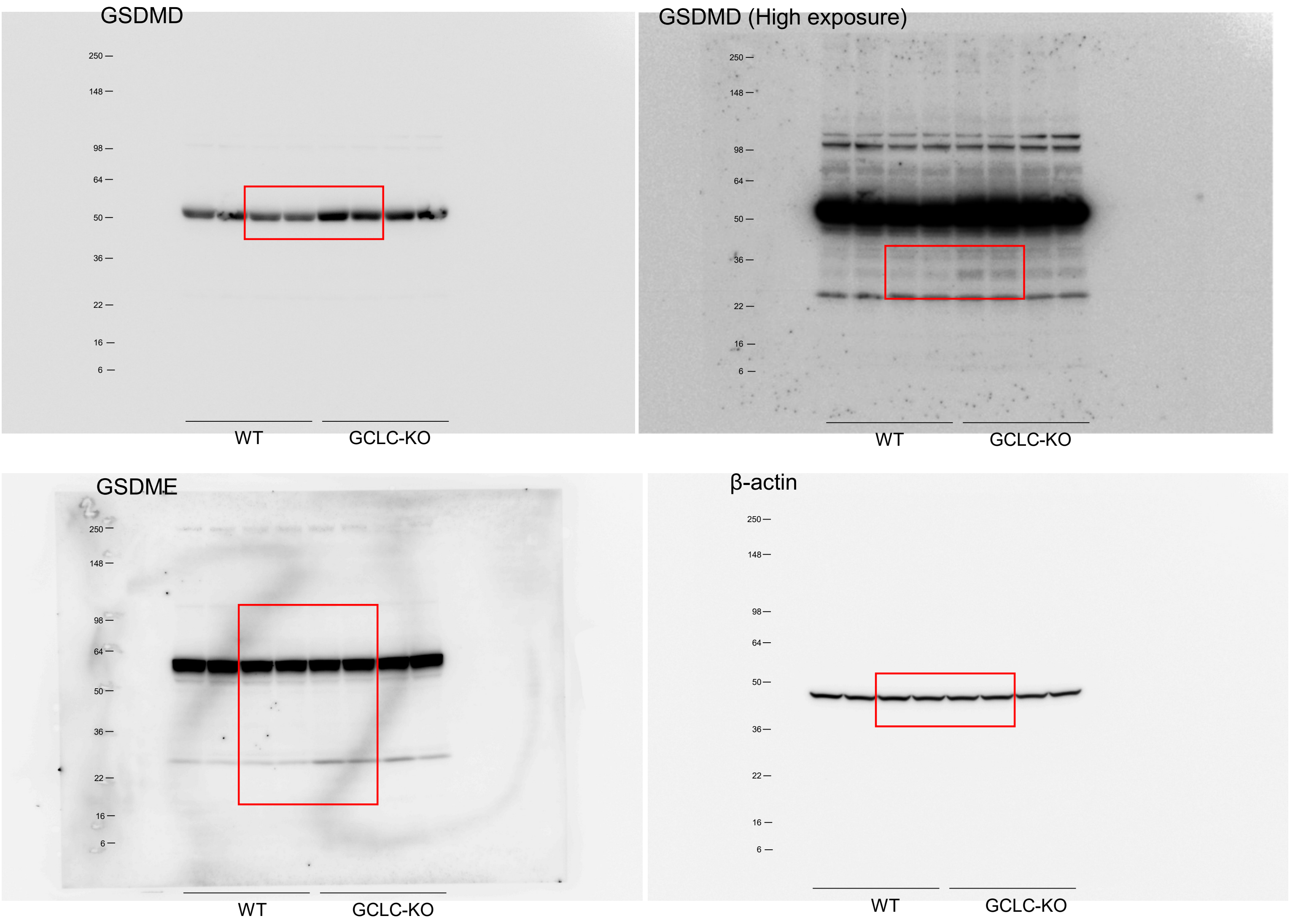

Figure S15 The full image for Figure 6A
Red frames are regions shown in Figure 6A.

Figure S16

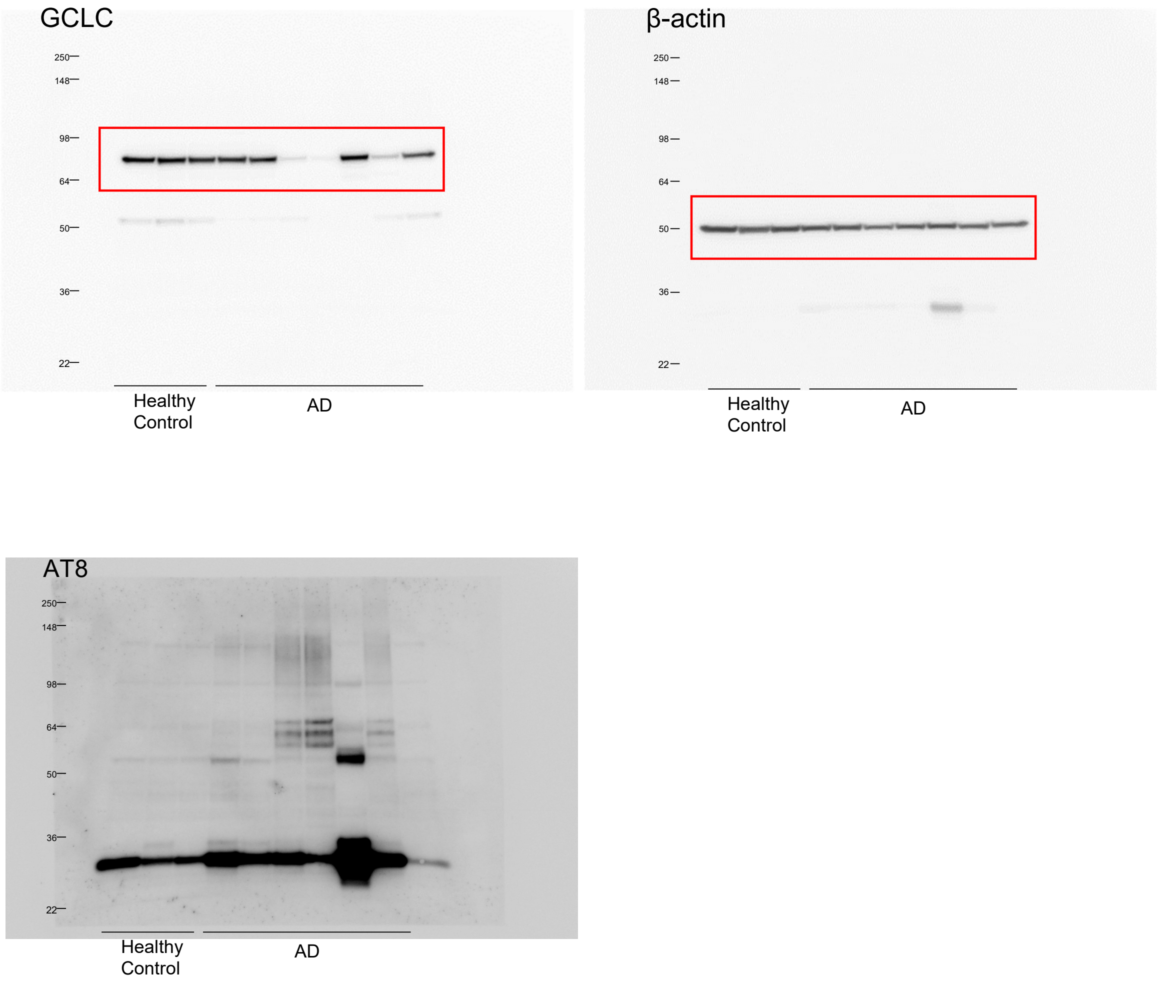

Figure S16 The full image for Figure 7C

Red frames are regions shown in Figure 7C. Western blot image of AD samples with AT8 antibody (phosphorylated-serine 202/phosphorylated-tyrosine 205 tau) is also shown.

Figure S17

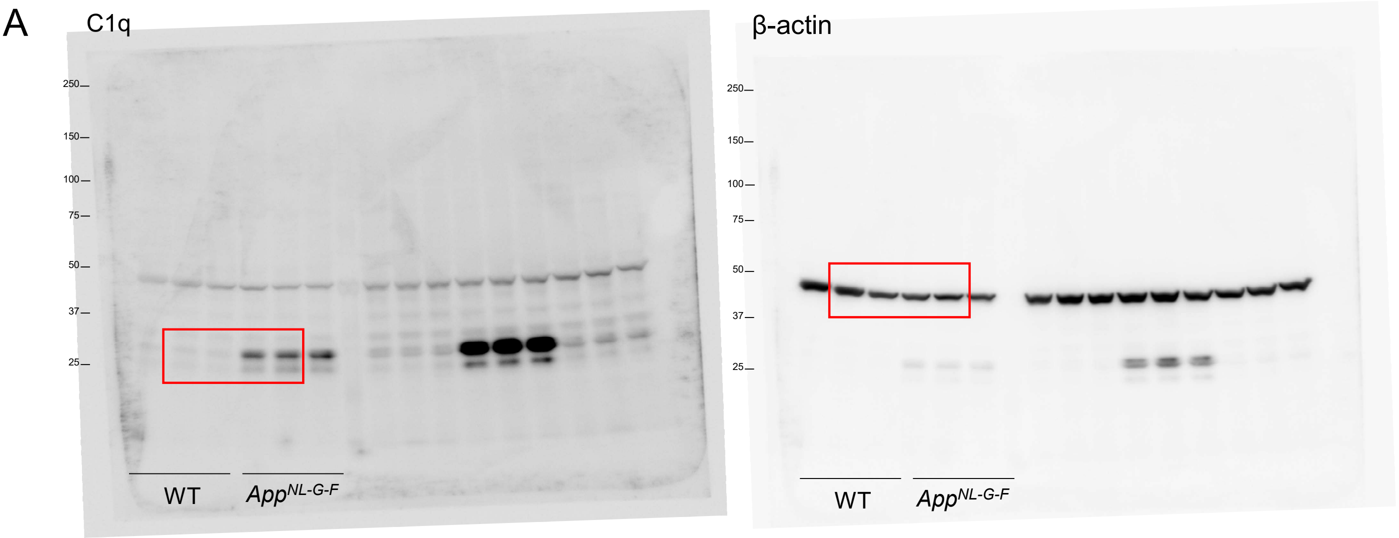

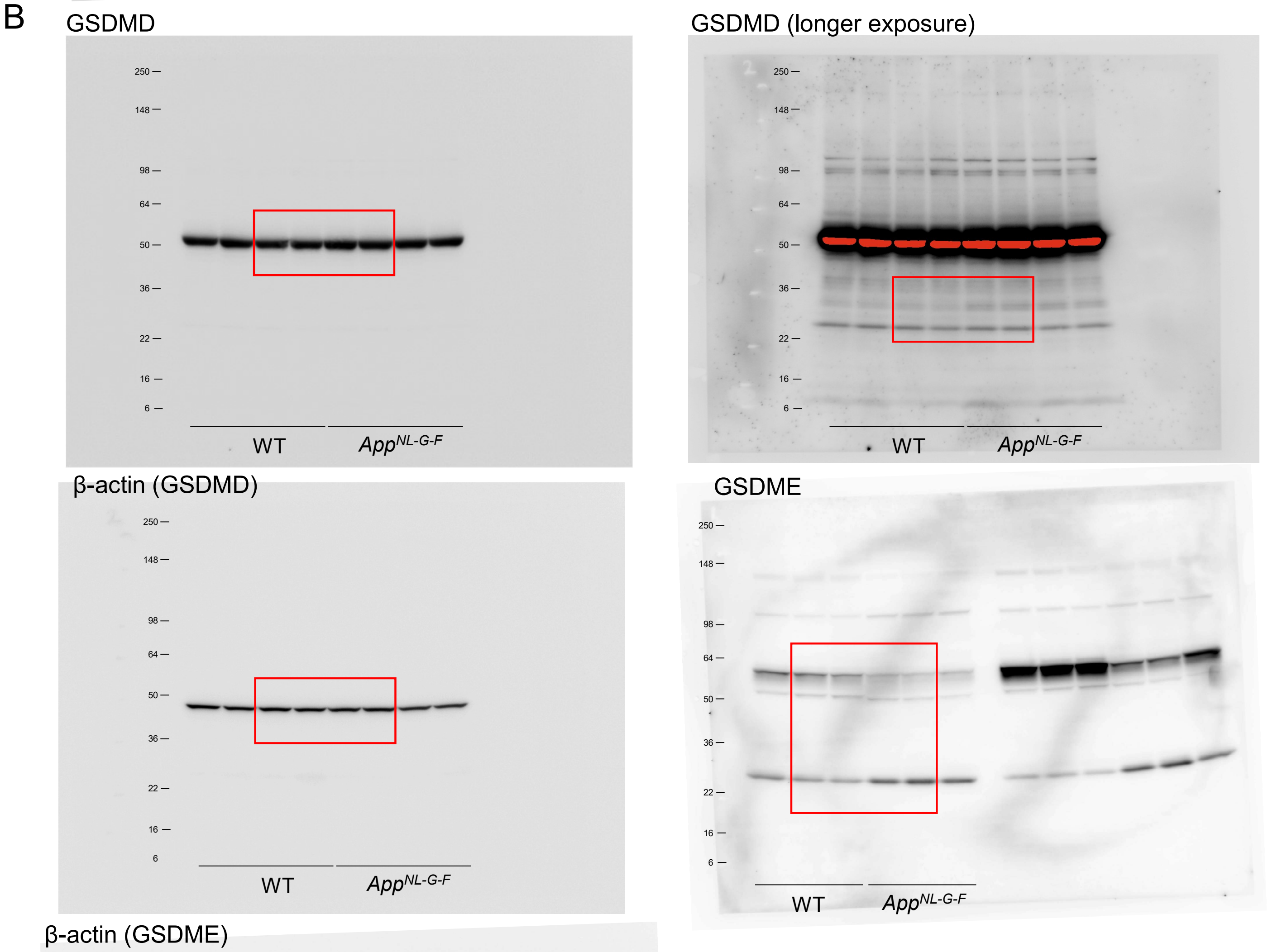

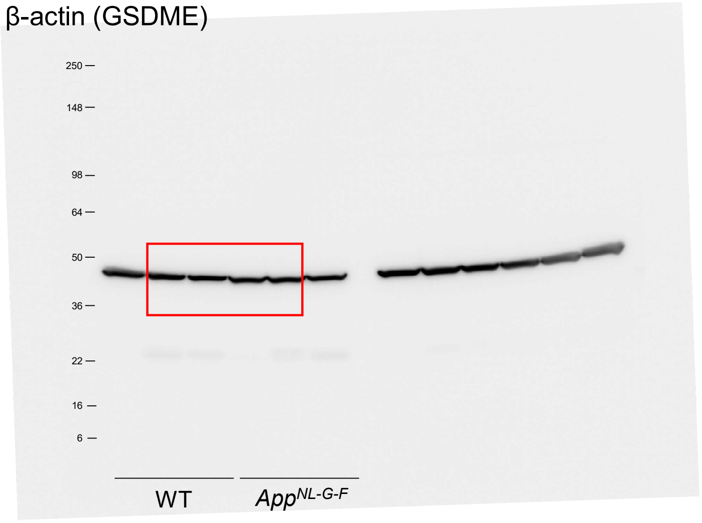

Figure S17 The full image for Figure 8
Red frames in A and B are regions shown in Figure 8D and 8E, respectively. Some images contain bands that are unrelated to this paper.
