# Supplementary material for: Neuronal glutathione loss leads to neurodegeneration involving gasdermin activation
Source: Sci Rep. 2023 Jan 20;13:1109. doi: 10.1038/s41598-023-27653-w (PMC9859798; doi:10.1038/s41598-023-27653-w)
Supplement: Supplementary file 3 — Supplementary Tables. [file 41598_2023_27653_MOESM3_ESM.pdf]

Table S1. Antibodies used for western blot (WB) and immunohistochemistry (IHC) analyses

The following antibodies were used at the indicated dilutions. Antigen activation and signal amplification methods in immunohistochemistry are shown in parentheses.

Protein	Antibody	Dilution WB	Dilution IHC (activation/amplification)
GCLC	Atlas Antibodies #HPA036359	1:5000	-
	Santacruz #sc390811	-	1:100 (Ac/TSA)
GCLM	Abcam #ab124827	1:2000	-
N1D (anti-Aβ)	Saido et al., Neurosci. Lett., 1996	-	1:200 (Ac/fluo-2nd Ab)
HO1	Enzo Life Sciences #SPA-895	1:1000	-
NeuN	Abcam #ab104224	-	1:2000 (Ac/Envision)
cleaved-caspase3	Cell Signaling #9661S	-	1:200 (Ac/TSA)
Iba1	Wako #013-27691	-	1:100 (Ac/direct)
	Abcam #ab5076	-	1:1000 (Ac/direct)
GFAP	Merck Millipore #MAB3402	-	1:100 (Ac/direct)
TSPO (PBR)	Abcam #ab109497	-	1:200 (Ac/TSA)
HMGB1	Abcam #ab18256	1:5000	-
C1q	Abcam #ab71089 (discontinued)	-	1:100 (Ac/Envision)
	Abcam #ab227072	1:2000	-
	Abcam #ab182451	-	1:500 (Protease/TSA)
C3	Novus #NB200-540	-	1:100 (- /direct)
PSD95	Synaptic System # 124011	-	1:1000 (Ac/TSA)
GSDMD	Abcam #ab219800	1:2000	-
GSDME (DFNA5)	Abcam #215191	1:5000	1:200 (Ac/TSA)
Homer 1	Synaptic Systems #160004	-	1:500(Ac/direct)
β-actin	Sigma #A5441	1:20000	-

Ac: autoclave

Protease: reaction with protease solution

direct: direct immunofluorescence method in which Alexa-conjugated 2nd antibodies are used

TSA: fluorescence-indirect tyramide signal amplification (TSA) system

Envision: DAKO EnVision+ system

Table S2. Primers used for RT-PCR analyses.

Gene name/direction	Sequence of primers
cst7 fw	ATGTCAGCAAAGCCCTGGTA
cst7 rv	GGTCTTCCTGCATGTAGTTCG
cx3cr1 fw	GGCCTAGAGCTCAAAGAAATCC
cx3cr1 rv	CACAGACCTTCGATCCCAGT
il1b fw	TGCCACCTTTTGACAGTGATG
il1b rv	TGATGTGCTGCTGCGAGATT
itgax fw	TCGTATTTGGCTTCCCAGAC
itgax rv	CCATCATTAGACACCGTCACAT
p2ry12 fw	CCCGGAGACACTCATATCCTT
p2ry12 rv	GTCCCAGGGGAGAAGGTG
trem2 fw	AGGGCCCATGCCAGCGTGTGGT
trem2 rv	CCAGAGATCTCCAGCATC
tyrobp fw	TGGTGTTGACTCTGCTGATTG
tyrobp rv	GTCTCAGCAATGTGTTGTTTCC
apoE fw	AAGCAACCAACCCTGGGAG
apoE rv	TGCACCCAGCGCAGGTA
cstb fw	TCTCCTACTTCTGATTCGGGG
cstb rv	GAATCACAACAGAAGCTGCTCAA
serpina3n fw	TGGCTGGTTTCAGCTCTGT
serpina3n rv	GTCAGTGTTGATGGAGGCCA
vim fw	TTCTCTGGCACGTCTTGACC
vim rv	CGATCTGGACATGCTGTTCCT
gata1 fw	TTTCATTCGCCCGGAGGTAG
gata1 rv	CAGGGTTCCAACTCCCAGAG
gsm fw	ATGAGGAGCTGGGAGGAACT
gsm rv	GGTGCCACCCTTGTAGATGA
osmr fw	AGCACAAACCTGAGCCTTGA
osmr rv	CCTTGACCTCTTCGTGTCCA
gclc fw	GCCATGGGGCTGCTGT
gclc rv	ACATGTACTCCACCTCGTCA
gclm fw	CGCCTCCGATTGAAGATGGA
gclm rv	ATTGGGTTTTACCTGTGCCC
gpx4 fw	CATTGGTCGGCTGCGTGA
gpx4 rv	CGCGGGATGCACACATGG
gsr fw	ATGCCACGTGATCTCAGCAC
gsr rv	CTCAATGAGCAGCACACATACTG
gss fw	TCCAAGTGGTGACTTCCGTG
gss rv	TCCGTCTGAATGCCCACTTT
ho-1 fw	CCTTCCCGAACATCGACAGCC
ho-1 rv	GCAGCTCCTCAAACAGCTCAA

Table S3. Details of mouse strains used in experiments

Age: age in months, M/F: Male/ Female. "WT M2, F2" means "2 males and 2 females of WT were used in the experiment".

Main Figures			
**Figure #**	**age**	**sex**	
1A	4, 8, 14, 19	all mice	M
1B, C	3-11	all mice	M
1D	8	WT	M1, F3
		GCLC-KO	M2, F2
2A, D	5	all mice	F
2B, C	3, 4, 8, 19	all mice	F
2E-G	4	all mice	F
3A	4	WT	M2, F2
		GCLC-KO	F4
3B	4	WT	M1, F3
		GCLC-KO	F4
4A, B	5	all mice	F
5	5	all mice	F
6A	8	WT	M1, F3
		GCLC-KO	M2, F2
6B	8	GCLC-KO	M2, F1
		GSDME-KO X GCLC-KO	M2, F1
6C, D	8	GCLC-KO	M2, F1
		GSDME-KO X GCLC-KO	M3
6E	4	GCLC-KO	M3, F1
		GSDME-KO X GCLC-KO	M1, F2
6F	3	all mice	F
7A	18	all mice	M
7B	24	all mice	F
7D	12	all mice	M
8A, B	12	all mice	M
8C	6	all mice	F
8D	6	all mice	F
8E	12	all mice (GSDMD)	M2, F2
	12	all mice (GSMDE)	F

Supplementary Figures			
**Figure #**	**age**	**sex**	
S1	3	all mice	M
S2A	6	all mice	F
S2B	3-11	GCLC-KO	M
S2B	5-7	WT	M
S2C	8,14,19	WT	F
S2C	4, 8, 14, 19	GCLC-KO	F
S3	14	all mice	F
S4	7	all mice	M
S5	8		M1, F1
S7	3	all mice	F
	8	all mice	M
S9A	8	GCLC-KO	F3
		GSDMD-KO X GCLC-KO	M1, F2
S9B	4	GCLC-KO	M3, F1
		GSDMD-KO X GCLC-KO	M2, F2
S10	4	GCLC-KO	M3, F1
		GSDME-KO X GCLC-KO	M1, F2
S11	8	WT	M2, F1
		GSDME-KO	M2, F1
S12	24	all mice	F
